# The onset of rare earth metallosis begins with renal gadolinium-rich nanoparticles from magnetic resonance imaging contrast agent exposure

**DOI:** 10.1038/s41598-023-28666-1

**Published:** 2023-02-04

**Authors:** Joshua DeAguero, Tamara Howard, Donna Kusewitt, Adrian Brearley, Abdul-Mehdi Ali, James H. Degnan, Stephen Jett, John Watt, G. Patricia Escobar, Karol Dokladny, Brent Wagner

**Affiliations:** 1 Kidney Institute of New Mexico, University of New Mexico Health Science Center, Albuquerque, NM USA; 2 University of New Mexico Health Science Center, Albuquerque, NM USA; 3 New Mexico Veterans Administration Health Care System, Albuquerque, NM USA; 4 Department of Earth and Planetary Sciences, University of New Mexico, Albuquerque, NM USA; 5 Department of Mathematics and Statistics, University of New Mexico, Albuquerque, NM USA; 6 Chan Zuckerberg Initiative, Redwood City, CA USA; 7 Center for Integrated Nanotechnologies, Los Alamos National Laboratory, Los Alamos, NM 87545 USA

**Keywords:** Acute kidney injury, Experimental models of disease, Metals

## Abstract

The leitmotifs of magnetic resonance imaging (MRI) contrast agent-induced complications range from acute kidney injury, symptoms associated with gadolinium exposure (SAGE)/gadolinium deposition disease, potentially fatal gadolinium encephalopathy, and irreversible systemic fibrosis. Gadolinium is the active ingredient of these contrast agents, a non-physiologic lanthanide metal. The mechanisms of MRI contrast agent-induced diseases are unknown. Mice were treated with a MRI contrast agent. Human kidney tissues from contrast-naïve and MRI contrast agent-treated patients were obtained and analyzed. Kidneys (human and mouse) were assessed with transmission electron microscopy and scanning transmission electron microscopy with X-ray energy-dispersive spectroscopy. MRI contrast agent treatment resulted in unilamellar vesicles and mitochondriopathy in renal epithelium. Electron-dense intracellular precipitates and the outer rim of lipid droplets were rich in gadolinium and phosphorus. We conclude that MRI contrast agents are not physiologically inert. The long-term safety of these synthetic metal–ligand complexes, especially with repeated use, should be studied further.

## Introduction

The properties of magnetic resonance imaging (MRI) contrast agents rely on a rare earth metal, gadolinium. Because gadolinium is toxic, magnetic resonance imaging contrast agents are proprietary aminopolycarboxylic acid chelates designed to bind the metal tightly and enhance renal elimination. Complications of MRI contrast agents include (sometimes fatal) gadolinium encephalopathy, acute kidney injury, gadolinium deposition disease/symptoms associated with gadolinium exposure (SAGE)^1^, and ‘nephrogenic’ systemic fibrosis^1–5^. Exposure to any class of MRI contrast agent leads to the long-term retention of gadolinium^6^. Residual gadolinium from MRI contrast agent exposure has been found in every vital organ, including the brain, in both patients and animal models^7–11^. Urine can contain gadolinium years after exposure to MRI contrast agents^12^.

Our rodent models demonstrated the formation of gadolinium-rich nanoparticles in the kidney and skin following systemic MRI contrast agent treatment^13–19^. Gadolinium-rich densities have been found in neuronal cytoplasm and nuclei in the brains of individuals exposed to MRI contrast agents during the course of routine care^11^. The nanotoxicologic mechanisms of gadolinium-induced disease are poorly understood^5,13–20^. Our understanding of MRI contrast-induced complications is far from complete. These studies were conducted to characterize the composition of intracellular gadolinium-rich minerals that form after systemic MRI contrast agent treatment.

## Results

### Magnetic resonance imaging (MRI) contrast agent treatment induced pathologic changes in the skin, liver, and kidney

Mice were treated with gadolinium-based contrast agent according to our established protocols^13–18,20,21^. Skin changes, including fibrosis, increased dermal cellularity, and epidermal thickening (Supplementary Fig. S1a–d), were like what we have previously reported^15,16,20,21^. There was vacuolation of renal cortical tubular epithelium from gadolinium-treated mice (Supplementary Fig. S1e). These findings are similar to what we have previously reported in rodent models of gadolinium-induced renal damage^14–17,20,21^.

### MRI contrast agent treatment induced renal pathologic changes in the male and the female mouse groups

**Figure 1:**
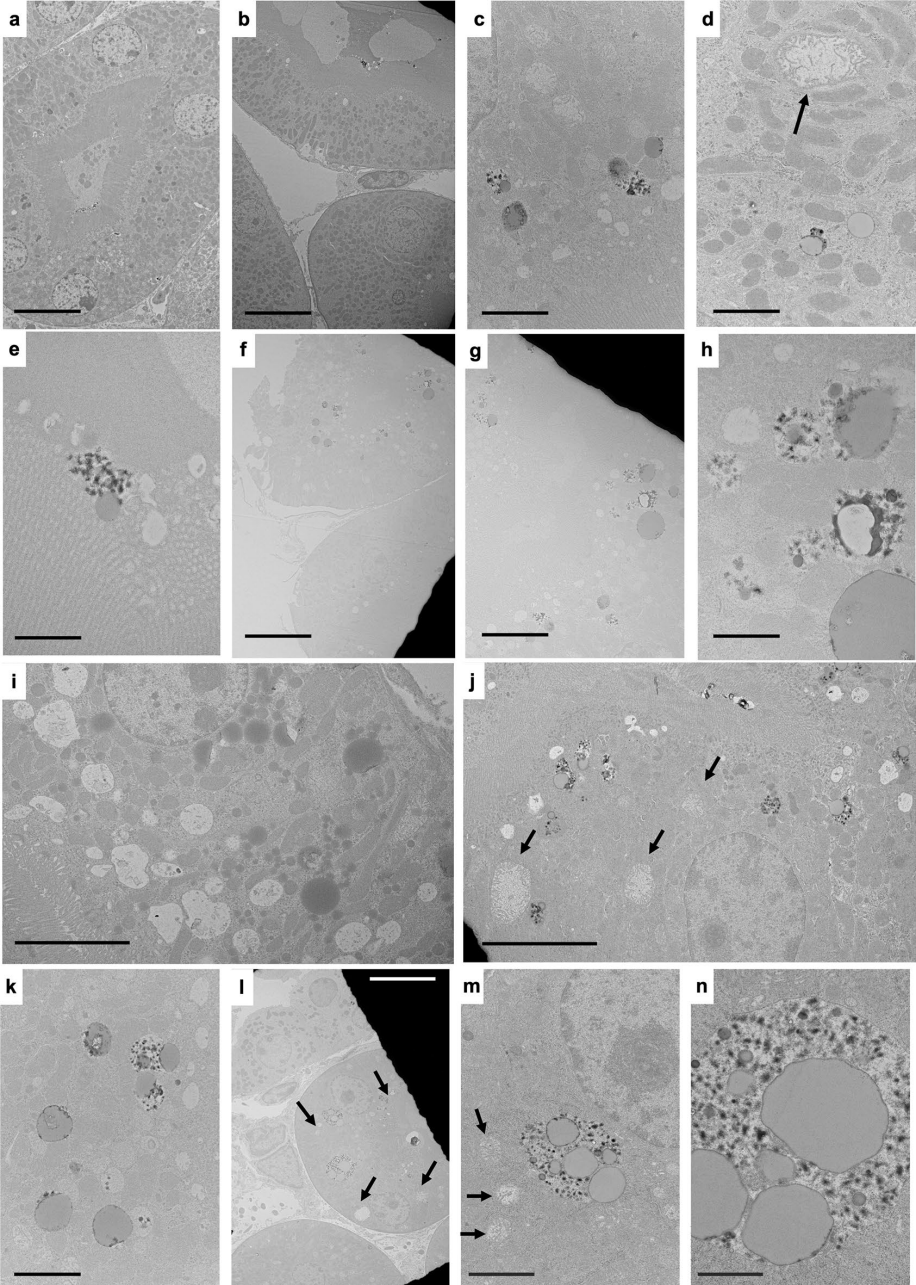
Renal proximal tubular changes in MRI contrast agent-treated female mice. (**a**) Renal proximal tubular cell of untreated female, showing a normal dense brush border and histologically-normal round nuclei. Calibration bar = 10 µm. (**b**) Ruptured basement membrane of a renal proximal tubular tubule in a MRI contrast agent-treated female. Calibration bar = 10 µm. (**c**) Proximal tubules contained many lipid-laden vacuoles with electron-dense borders, dysmorphic mitochondria, and increased cytoplasmic vacuoles (many with lipid-like bodies and electron-dense precipitates). Calibration bar = 2 µm. (**d**) Proximal tubule harboring a large, toxic mitochondrion (arrow), lipid-laden vacuoles (often rimmed with electron-dense material), from a MRI contrast agent-treated female. Calibration bar = 2 µm. (**e**) Spiculated, sea urchin-shaped electron densities adjacent to a lipid droplet outside the brush border of a renal proximal tubule. Calibration bar = 1 µm. (**f**) Renal proximal tubules with atypical nuclei, lipid-laden vacuoles (again, rimmed with electron-dense material), from a MRI contrast agent-treated female. Calibration bar = 10 µm. (**g**) Magnification of brush border from (**f**). Note the large lipid droplets and electron-dense nanostructures. Bar = 5 µm. (**h**) Magnified region from (**g**), highlighting electron densities surrounding lipid droplets and mitochondria. Calibration bar = 1 µm. (**i**) Increased cytoplasmic vacuolation within the renal proximal tubule and bloated mitochondria from a gadolinium-treated female. Calibration bar = 5 µm. (**j**) Mitochondriopathy (arrows), characterized by matrix expansion and cristae loss in the renal proximal tubule from a treated female. The cell also contains vacuolized lipid concomitant with electron-dense nanoparticles. Calibration bar = 5 µm. (**k**) Unilamellar vesicles within renal epithelium from gadolinium-treated animals frequently are rimmed with electron-dense material and sometimes coincide with sea urchin-appearing, spiculated nanostructures. Calibration bar = 2 µm. (**I**) Proximal tubule cells with large cytoplasmic vesicles containing lipid and electron-dense precipitates. Also featured is an increase in interstitial cellularity from a gadolinium-treated female. Calibration bar = 10 µm. (**m**) Magnification of the complex cytoplasmic vacuole in (**l**). The arrows indicate mitochondriopathy. Calibration bar = 2.0 µm. (**n**) Magnification of a vesicle in (**l**) showing large lipid droplets and endocytosed electron-dense nanoparticles. Calibration bar = 1 µm. Hitachi H7700 TEM, AMT 16-megapixel digital camera.

At the ultrastructural level, renal glomerular and tubular pathologies were evident in the treated mice (Supplementary Fig. S2). Electron-dense material was a common feature in the kidney in males and females (Fig. 1). The electron-dense precipitates were dispersed throughout kidney sections and often rimmed large unilamellar vesicles (Supplementary Fig. S2b–d, g–j, Fig. 1c–h, j–n). Mitochondrial toxicity, characterized by swelling and an increased matrix-to-cristae ratio, was a common finding in gadolinium-treated males (Supplementary Fig. S2e–f, g, i) and females (Fig. 1c, d, j, m). Renal proximal tubules from gadolinium-treated males and females demonstrated increased numbers of enlarged cytoplasmic vesicles (Fig. 1g–j), apical blebbing (Supplementary Figs. S2j, S1d, I, j), tubular damage (Supplementary Fig. S2j), reduced mitochondrial density (Supplementary Fig. S2k, l), basement membrane rupture (Supplementary Fig. S2m), and occasionally rupture of the apical membranes (Supplementary Fig. S2n). Quantified morphometry from transmission electron microscopy is provided in Table 1.

**Table 1:** Electron microscopic morphometry of renal cortical intracellular organelles^*^

Cell	Organelle	Untreated	Treated	*P*	Adjusted *P*
Proximal tubule	Nuclei	24.5 ± 9.9	50.4 ± 110.4	0.09	0.2
Distal tubule		28.0 ± 4.0	15.9 ± 7.0	< 0.001	< 0.001
Interstitial		11.8 ± 8.2	9.4 ± 4.9	0.2	0.2
Proximal tubule	Mitochondria	0.9 ± 0.6	0.8 ± 1.9	0.4	0.5
Distal tubule		0.8 ± 0.6	0.7 ± 1.0	0.2	0.2
Proximal tubule	Cytoplasmic vesicles	0.3 ± 0.7	1.2 ± 3.5	< 0.001	< 0.001
Distal tubule		0.4 ± 0.2	0.5 ± 0.3	0.5	0.5

*Areas are in units of µm^3^. The Benjamini–Hochberg procedure derived false discovery rate-adjusted *P* values.

### Gadolinium treatment affected renal glomeruli (Supplementary Fig. S3), distal tubules, and interstitia in mice (Supplementary Fig. S4)

Renal glomerular parietal cells of gadolinium-treated mice demonstrated vacuolization (Supplementary Fig. S3e, f), sometimes with unilamellar vesicles (Supplementary Fig. S3g–i). Occasionally, podocytes showed similar abnormalities (Supplementary Fig. S3j). Distal tubular epithelial cells occasionally contained electron-dense material within vacuoles and signs of mitochondrial stress (Supplementary Fig. S4). Distal tubular epithelia also demonstrated intracellular unilamellar vesicles, occasionally rimmed with electron-dense material (Supplementary Fig. S4d). Interstitial expansion and increased cellularity with occasional vacuolized electron-dense material were present in the gadolinium-treated groups (Supplementary Fig. S4e–h).

Concomitant with the Warburg effect in the kidney, systemic gadolinium-based contrast agent treatment induces dyslipidemia and insulin resistance^14^. The impact of gadolinium-based contrast agent treatment in the liver was examined (Supplementary Fig. S5). Gadolinium increased intracellular triglycerides as assessed by oil red O staining. Electron microscopy revealed that gadolinium treatment increased unilamellar vesicles and reduced mitochondrial volume. Metabolomic analysis of the livers demonstrated alterations in metabolites associated with amino acid metabolism, glycogenesis, and glycolysis (Supplementary Table S1). These findings support the hypothesis that gadolinium-based contrast agents are not biologically inert^5^.

### Electron-dense material in kidney cells from MRI contrast agent-treated mice contained gadolinium

**Figure 2:**
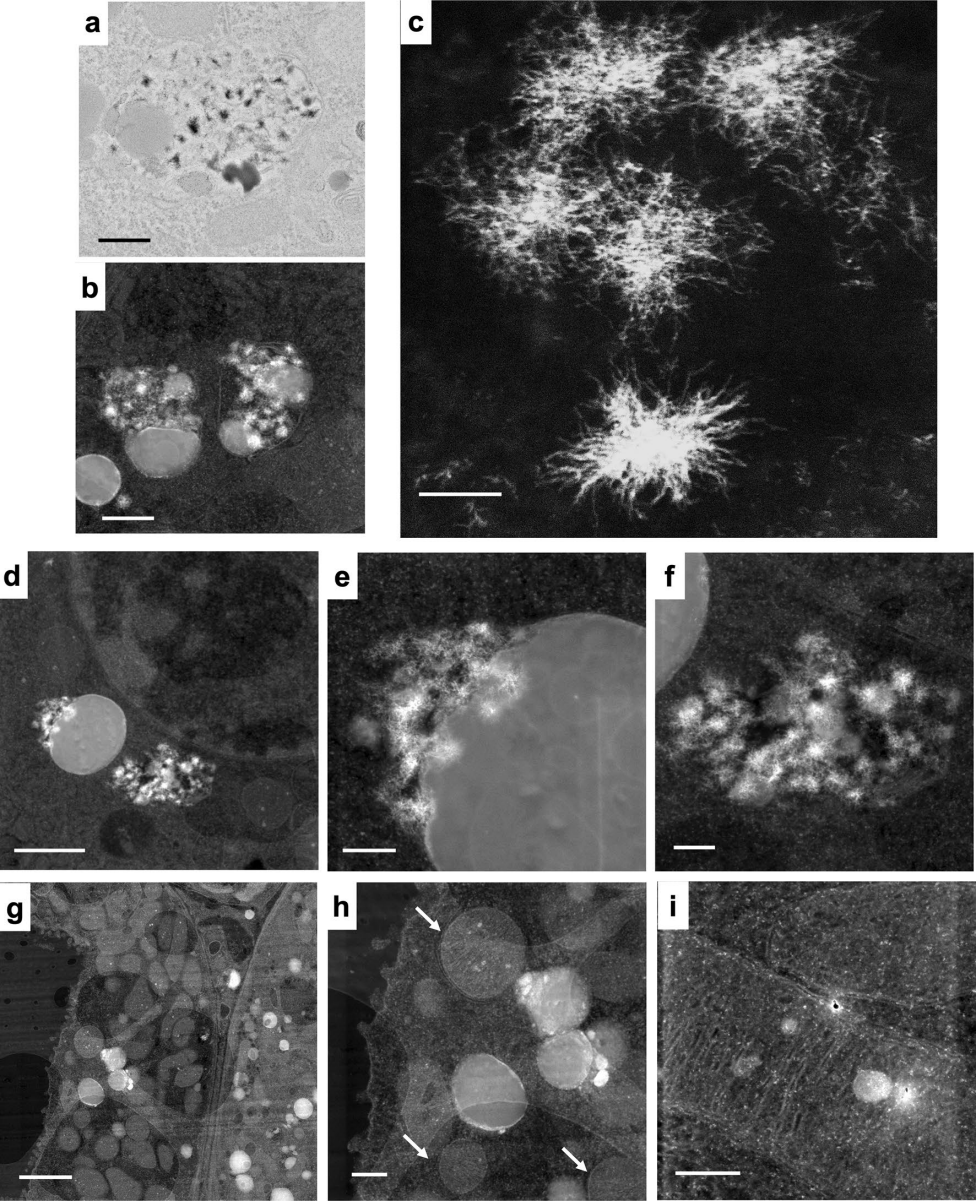
Spiculated electron-dense nanoparticles in the kidney arise from MRI contrast agent treatment. (**a**) Transmission electron micrograph of electron-dense spiculated material in a cytoplasmic vesicle, renal proximal tubule cell, from a MRI contrast agent-treated female. The vacuole also contains unilamellar (lipid) vesicles. Calibration bar = 500 nm. Hitachi H7700 TEM, AMT 16-megapixel digital camera. (**b**) Darkfield scanning transmission electron microscopic image of cytoplasmic vesicles containing lipid droplets with electron-dense nanoparticles from a MRI contrast agent-treated male. Calibration bar = 500 nm. FEI Tecnai G(2) S-Twin (300 kV) transmission electron microscope equipped with an EDAX ECON X-ray detector. (**c**) High magnification of filamented, spiculated, electron-dense nanoparticles in the kidney from a MRI contrast agent-treated male. Scanning transmission electron microscopy. Calibration bar = 50 nm. JEOL 2010F FEGSTEM 200 kV transmission electron microscope with silicon drift detector. (**d**) Peri-nuclear unilamellar vesicle and spiculated nanoparticles in a renal epithelial cell from a MRI contrast agent-treated female. Scanning transmission electron microscopy. Calibration bar = 1 µm. (**e**) Magnification of the area in (**d**). Calibration bar = 200 nm. (**f**) Magnification of electron-dense nanoparticles in (**d**). Scanning transmission electron microscopy. Calibration bar = 200 nm. (**g**) Dark field scanning transmission electron microscopy image of kidney cortex from a MRI contrast agent-treated male. Multiple intracellular unilamellar vesicles, electron-dense material, and mitochondria are visible. Calibration bar = 2 µm. (**h**) Magnification of the region from (**g**) demonstrates rounded mitochondria (arrows) and lipid bodies rimmed with electron-dense material. Calibration bar = 500 nm. (**i**) A mitochondrion in the renal cortex with electron-dense inclusion, from a MRI contrast agent-treated male. Scanning transmission electron microscopy. Calibration bar = 250 nm. (**d**–**i**), FEI Tecnai G(2) S-Twin (300 kV) transmission electron microscope equipped with an EDAX ECON X-ray detector.

Gadolinium can be detected in several organs after systemic treatment with MRI contrast agents^18^. The electron-dense precipitates and electron-dense material rimming unilamellar vesicles/lipid droplets were localized using transmission electron microscopy (Fig. 2). These regions were then identified using a STEM equipped with XEDS (Fig. 2b–i). The spiculated, sea-urchin-like intracellular precipitates were visualized in darkfield mode from specially sectioned and mounted tissues (Fig. 2c). Electron-dense material rimming vacuolized lipid droplets (Fig. 2d–e) and spiculated nanostructures (Fig. 2f) were identifiable by Z-contrast. In addition to the pathologic electron-dense material, mitochondria and cellular nuclei could often be visualized (Fig. 2g, h). Electron-dense regions were occasionally found within the mitochondria of treated animals (Fig. 2i).

### Intracellular gadolinium-rich material correlated with high phosphorus concentrations in mice

**Figure 3:**
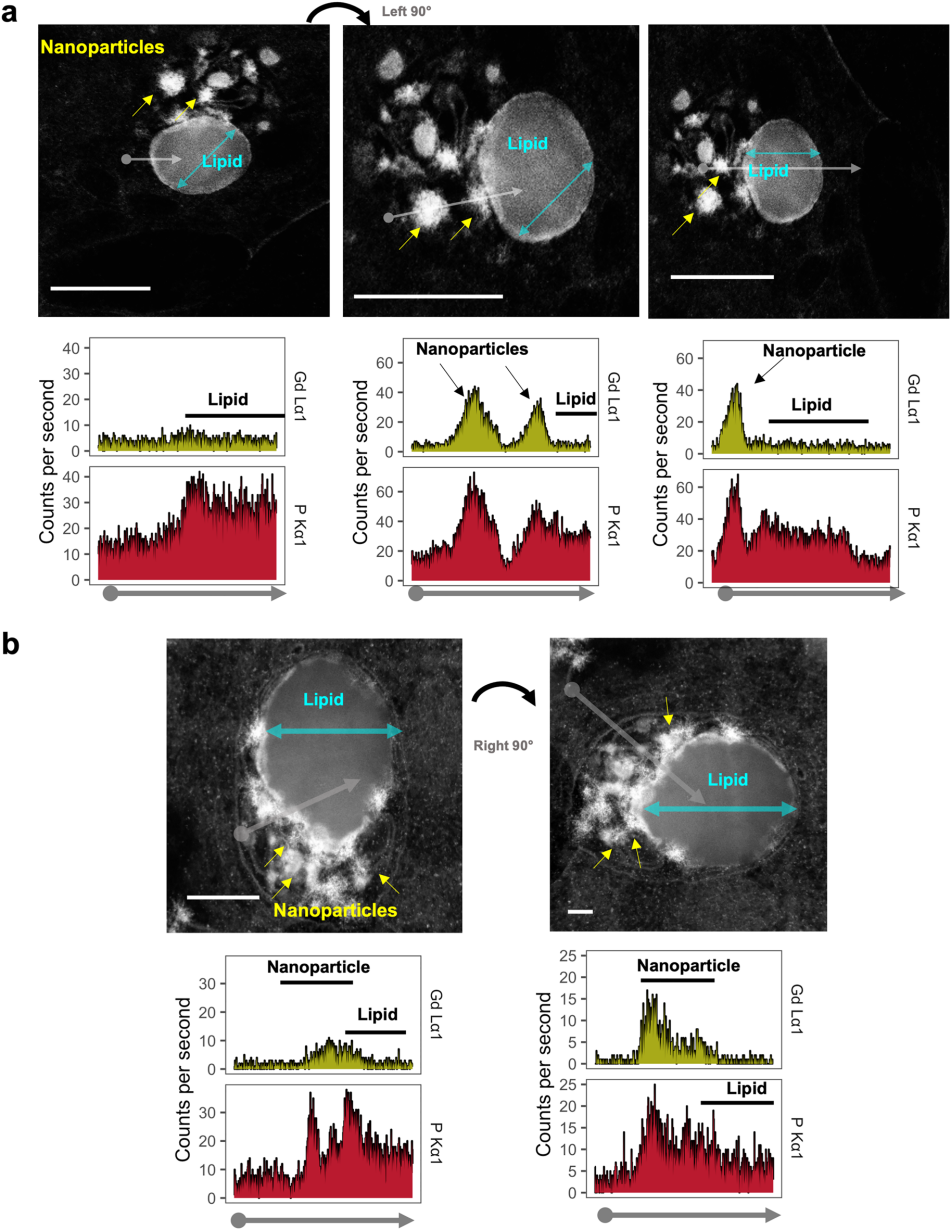
Energy-dispersive X-ray spectroscopy (XEDS) line scan profiles through sub-cellular regions. Kidney samples were obtained from a MRI contrast agent-treated male mouse. (**a**) (Left) EDS line scan through intracellular lipid droplet avoiding electron-dense precipitates (arrows). XEDS line scan data (X-ray intensity in counts per second vs distance) for phosphorus (P) corresponding to the non-lipid and unilamellar vesicular regions. (Middle panel) Area on the left rotated to illustrate an XEDS line scan profile (arrow), through precipitates (arrowheads), and a unilamellar vesicle. The nanoparticles exhibited high amounts gadolinium (Gd) and phosphorus (P). (Right) XEDS line scan data through a single electron-dense nanoparticle and unilamellar vesicle. Electron-dense precipitates had high amounts of gadolinium and phosphorus. Bars = 2.5 µm. (**b**) (Left) XEDS line scan data through electron-dense nanoparticles (arrows), and unilamellar vesicle from a MRI contrast-agent-treated male, and corresponding amounts of gadolinium and phosphorus. (Right) XEDS line scan (grey arrow) through the cytoplasm, vacuole membrane, electron-dense precipitate, and unilamellar vesicle. Bars = 0.1 µm. Corresponding line scan data for elements of interest from the cytoplasm, nanoparticles, and unilamellar vesicle. JEOL 2010F FEGSTEM 200 kV transmission electron microscope, with Oxford Analytical AZTec XEDS system, equipped with XMax 80 N 80 mm^2^ silicon drift detector.

**Figure 4:**
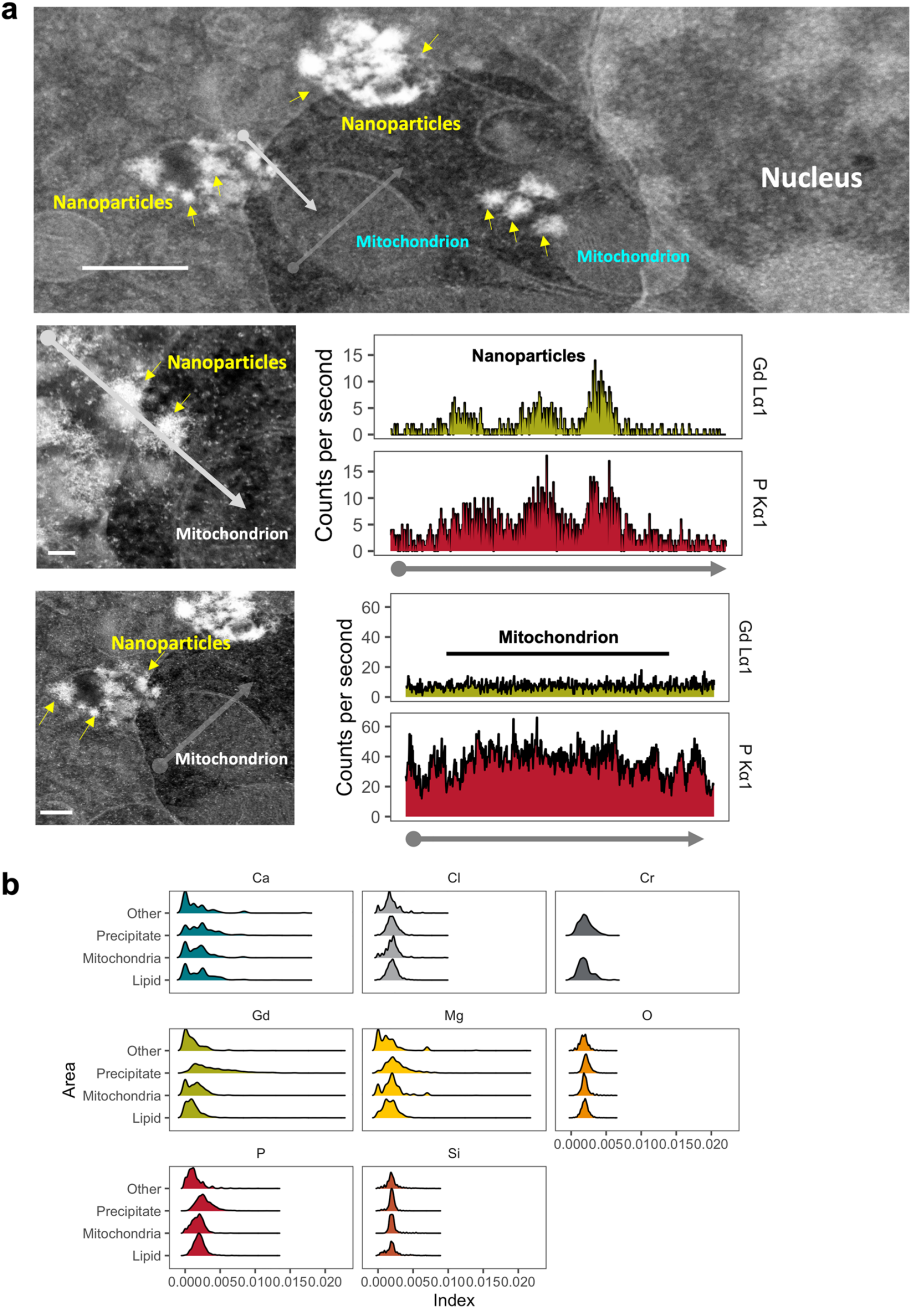
Elemental composition of nanoparticles differs from other subcellular regions. Renal cortex from a MRI contrast-agent treated mouse was analyzed by darkfield STEM. (**a**) (Top panel) Clusters of electron-dense precipitates identified in an epithelial cell from kidney cortex of a MRI contrast agent-treated male mouse. The numerous mitochondria and ellipsoid nucleus suggest a proximal tubular cell. Bar = 0.5 µm. (Middle panel) XEDS line scan through several spiculated electron-dense nanoparticles and mitochondrion. Bar = 50 nm. The XEDS line scan data (X-ray intensity in counts per second vs distance) shows high levels of gadolinium and phosphorus corresponding with the line scan through the nanoparticles. (Lower panel) Mitochondria and cytoplasm do not show elevations in gadolinium. An XEDS line scan through a mitochondrion and cytoplasm (avoiding electron-dense precipitates). Calibration bar = 0.2 µm, JEOL 2010F FEGSTEM 200 kV transmission electron microscope, with Oxford Analytical AZTec XEDS system, equipped with XMax 80 N 80 mm^2^ silicon drift detector. (**b**) Ridgeline plots for calcium (Ca), chlorine (Cl), chromium (Cr), gadolinium (Gd), magnesium (Mg), oxygen (O), phosphorus (P), and silicon (Si) in intracellular precipitates, unilamellar bodies/lipids, mitochondria, and other subcellular regions. The XEDS signals were indexed to the total areas under each curve.

Gadolinium is not a normal trace element^13^ and possesses a signature signal (particularly in the L electron shell energy range) detectable by XEDS^18^. The chemical compositions of these electron-dense materials were assessed in many subcellular regions via XEDS (Supplementary Figs. S6, S7, S8). XEDS line scan data were obtained for gadolinium, phosphorus, calcium, chlorine, chromium, magnesium, oxygen, and silicon. Electron-dense precipitates contained gadolinium and phosphorus (Fig. 3, Supplementary Figs. S6, S7, S8). Non-precipitate regions and the centers of lipid droplets did not contain gadolinium (Supplementary Figs. S6, S7, S8), and the electron-dense material rimming lipids (Supplementary Figs. S6b, S7e, f). Mitochondria tended to have low gadolinium concentrations (Supplementary Fig. S9, Fig. 4). The XEDS line scan data of the subcellular regions (Supplementary Fig. S9, Fig. 4) revealed that gadolinium concentrations differed among electron-dense precipitates from mitochondria, lipid, and non-mitochondrial/non-lipid regions (*P* = 0) (Fig. 4b, Supplementary Table S2). Concomitantly, phosphorus (*P* < 1 × 10^–5^), calcium (*P* < 3 × 10^–9^), magnesium (*P* = 0), manganese (*P* = 0), and sulfur (*P* = 0.001) in the precipitate differed from these subcellular regions (Supplementary Figs. S9, S10). Linear regression for gadolinium and phosphorus signal intensities showed the strongest correlation between the 2 in precipitates (multiple *r*^*2*^ of 0.22, 0.25 for females and males, respectively; *P* < 0.001 by ordinary least squares).

### The electron-dense precipitates' concordance of gadolinium with phosphorus in mice was confirmed with 2D XEDS (Fig. 5)

**Figure 5:**
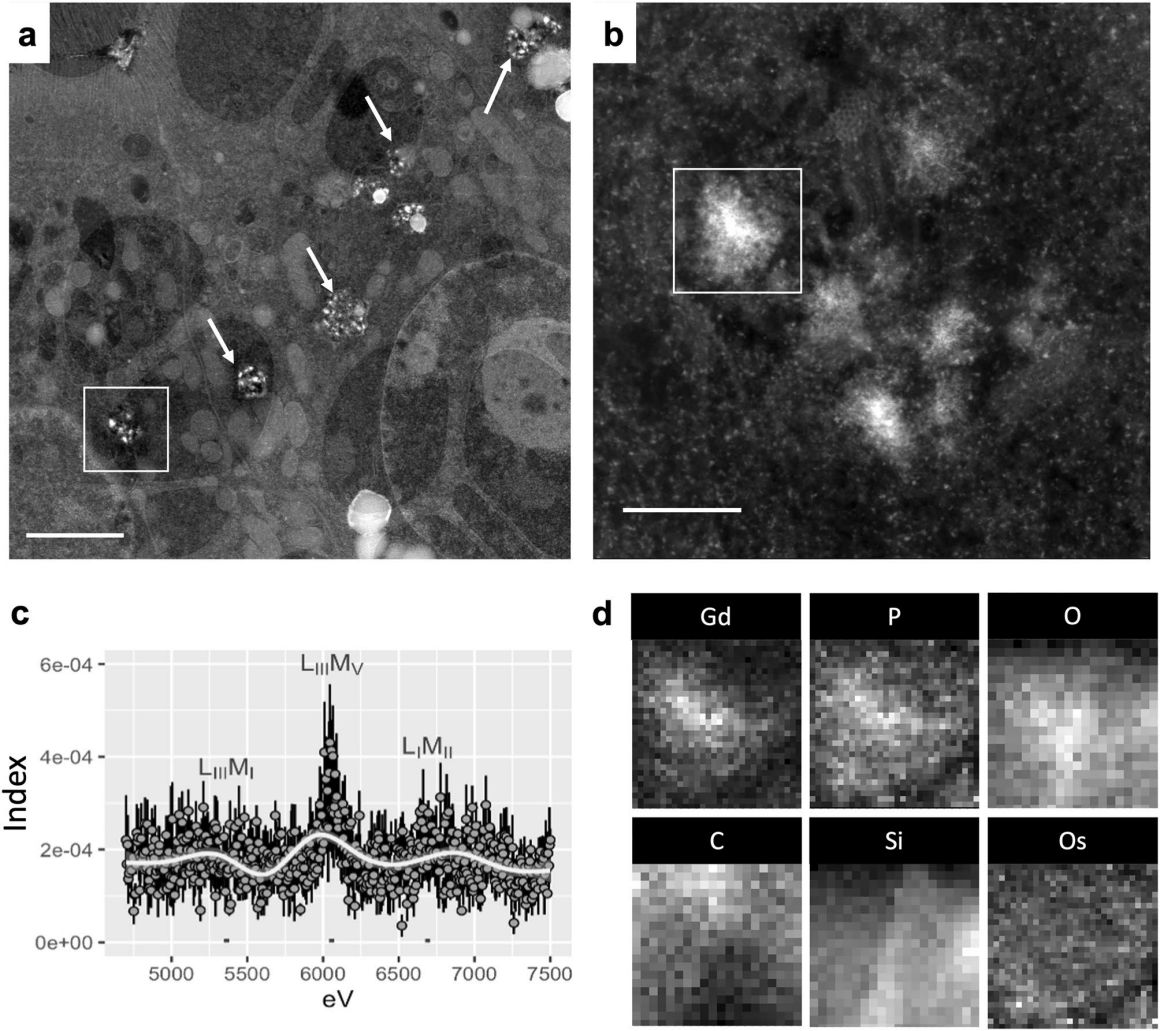
MRI contrast agent-induced nanoparticles are gadolinium rich. Renal cortex was analyzed from a MRI contrast agent-treated mouse. (**a**) Intracellular electron-dense spiculated nanoparticles pepper renal tubular cells (arrows). Bar = 2 μm. (**b** Magnified region from (**a**) showing an intracellular cluster of electron-dense, sea urchin-shaped precipitate. Bar = 200 nm. (**c**) XEDS data of the precipitate, gadolinium L energy range (L_III_M_I_, 5.362 eV; L_III_M_V_, 6.058 eV; and L_I_M_II_, 6.690). The L electron shell energies are far from those of physiologic elements, lending these signals to be specific for gadolinium^18^. Mean ± SE, *n* = 4 individual precipitates. (**d**) 2-dimensional (2D) XEDS map for gadolinium (Gd), phosphorus (P), oxygen (O), carbon (C), silicon (Si), and osmium (Os) of the electron-dense nanostructure featured in panel (**b**). Tecnai F30 300kv transmission electron microscope equipped with an EDAX detector.

Regions rich in electron-dense nanostructures were mapped (Fig. 5a, b) using STEM and XEDS. XEDS signals from precipitates in the gadolinium L electron shell energy ranges were non-zero (Fig. 5c). Two-dimensional mapping demonstrated the colocalization of gadolinium with phosphorus (Fig. 5d). Secondary 2D XEDS mapping of the electron densities confirmed that these nanoparticles were rich in gadolinium and phosphorus (Supplementary Fig. S11). Outside of the nanoparticles, other subcellular regions contained little or no gadolinium (Fig. 4, Supplementary Fig. S6).

From the mouse tissues, multiple variable linear modeling of the XEDS line scan data was used to analyze the elemental composition of the subcellular regions (i.e., gadolinium-rich nanoparticles, unilamellar/lipid-rich droplets, and mitochondria) relating gadolinium to the other assessed elements (Table 2). The quality of the model for electron-dense precipitates was optimized by the Akaike information criterion (AIC) method. The optimal model (Akaike information criteria, AIC) for the electron-dense debris correlated gadolinium to phosphorus and oxygen (Table 3). Principal component analysis supported the correlations of phosphorus and gadolinium in precipitates (Fig. S12). These data show that gadolinium de-chelates from MRI contrast agent formulations and precipitates intracellularly. This phenomenon is concomitant with lipid vacuolization, mitochondrial damage, and subacute tubular damage.

**Table 2:** Model 1 multivariable linear regression*.

Area	Element	Estimate	SE	*t* value	Pr (> (|*t*|)
Precipitate	(Intercept)	0.0006	0.0004	1.7	0.08
	P	1.0294	0.0481	21.3	< 2 × 10^–16^
	Ca	0.1147	0.0481	4.6	4 × 10^–6^
	Cl	−0.1579	0.0654	−2.4	0.02
	Cr	−0.0267	0.0543	−0.5	0.6
	Mg	0.0188	0.0269	0.7	0.5
	O	1.0471	0.1043	10.0	< 2 × 10^–16^
	Si	−0.8586	0.1119	−7.68	2 × 10^–14^
Lipid bodies	(Intercept)	−0.0001	0.0002	−0.8	0.4
	P	0.4015	0.0352	11.4	< 2 × 10^–16^
	Ca	0.0287	0.0151	1.9	0.06
	Cl	−0.0212	0.0343	−0.6	0.5
	Cr	0.0068	0.0324	0.2	0.8
	Mg	0.0227	0.0194	1.2	0.2
	O	0.4233	0.0570	7.4	1.6 × 10^–13^
	Si	−0.2114	0.0310	−6.8	1.2 × 10^–11^
Mitochondria	(Intercept)	0	0.0002	0.2	0.8
	P	0.4713	0.0433	10.9	< 2 × 10^–16^
	Ca	0.0792	0.0214	3.7	0.0002
	Cl	0.0491	0.0404	1.2	0.2
	Cr	0.0382	0.0394	1.0	0.3
	Mg	−0.0118	0.0266	−0.4	0.7
	O	0.0998	0.0693	1.4	0.2
	Si	0.0369	0.0623	0.1	0.6
Other	(Intercept)	0.0004	0.0001	3.1	0.002
	P	0.2499	0.0253	9.9	< 2 × 10^–16^
	Ca	0.0183	0.0121	1.5	0.1
	Cl	0.0087	0.0300	0.3	0.8
	Cr	0.0163	0.0307	0.5	0.6
	Mg	0.0313	0.0223	1.4	0.2
	O	0.0940	0.0466	2.0	0.04
	Si	0.0122	0.0301	0.4	0.7

*Model 1 was Gd ~ P + Ca + Cl + Cr + Mg + O + Si. Residuals (minimum, median, and maximum) for precipitate, lipid-rich bodies, mitochondria, and other were −0.007, −0.0005, 0.0144; −0.004, −0.0003, 0.0158; −0.004, −0.0001, 0.007; and −0.004, −0.0003, 0.01, respectively. Residual standard of error for electron-dense precipitates was 0.003 on 2265 degrees of freedom, multiple r^2^ 0.29, adjusted r^2^ 0.29, *F*-statistic 134.1 on 7 and 2265 degrees of freedom, *P* < 2.2 × 10^–16^. Residual standard error for lipid-rich bodies was 0.001 on 2475 degrees of freedom, multiple r^2^ 0.09, adjusted r^2^ 0.09, *F*-statistic 35.8 on 7 and 1036 degrees of freedom, *P* < 2.2 × 10^–16^. Mitochondrial residual standard error 0.001 on 1036 degrees of freedom, multiple r^2^ 0.14, adjusted r^2^ 0.14, *F*-statistic 24.8 on 7 and 1036 degrees of freedom, *P* < 2.2 × 10^–16^. For other (presumably intracellular) areas, residual standard of error was 0.001 on 2192 degrees of freedom, multiple r^2^ 0.05, adjusted r^2^ 0.05, *F*-statistic 16.9 on 7 and 2192 degrees of freedom, *P* < 2.2 × 10^–16^. The Akaike information criterion was −13,958 for precipitate, this model.

**Table 3:** Model 2 multivariable linear regression.*

Area	Element	Estimate	SE	*t* value	Pr(> (|*t*|)
Precipitate	(Intercept)	−0.001	0.0002	−5.4	9 × 10^–8^
	P	1.08	0.04	22.7	< 2 × 10^–16^
	O	1.07	0.12	10.2	< 2 × 10^–16^

*Model 2 was Gd ~ P + O (using intracellular precipitates only). Residuals (minimum, median, and maximum) for precipitate were −0.007, −0.0006, and 0.016. Residual standard error was 0.003 on 2270 degrees of freedom, multiple r^2^ 0.27, adjusted r^2^ 0.27, *F*-statistic 413.8 on 2 and 2270 degrees of freedom, *P* < 2.2 × 10^–16^. The Akaike information criterion for Model 2 was -20,625.

### MRI contrast agent use in humans leads to detectable gadolinium in the kidney

In humans, *permanent brain gadolinium retention* may occur from the routine use of MRI contrast agents^22^. The kidney is a reservoir for gadolinium in rodent models^13,14,18,20,23^. Therefore, we investigated the potential of lanthanide metallosis in humans. Human kidney samples were obtained from the University of New Mexico Human Tissue Repository. The Repository is accredited by the College of American Pathologists Guidelines for Biorepositories. There were equal numbers of MRI contrast agent-exposed and unexposed donors (*n* = 5 each). Gadolinium was quantitated with inductively coupled plasma mass spectroscopy (University of New Mexico Department of Earth & Planetary Sciences). Gadolinium was detectable in 100% of the samples where the donors had histories of MRI contrast agent exposure (Supplementary Fig. S13).

### Nanoparticles in human kidney are also primarily gadolinium and phosphorus

**Figure 6:**
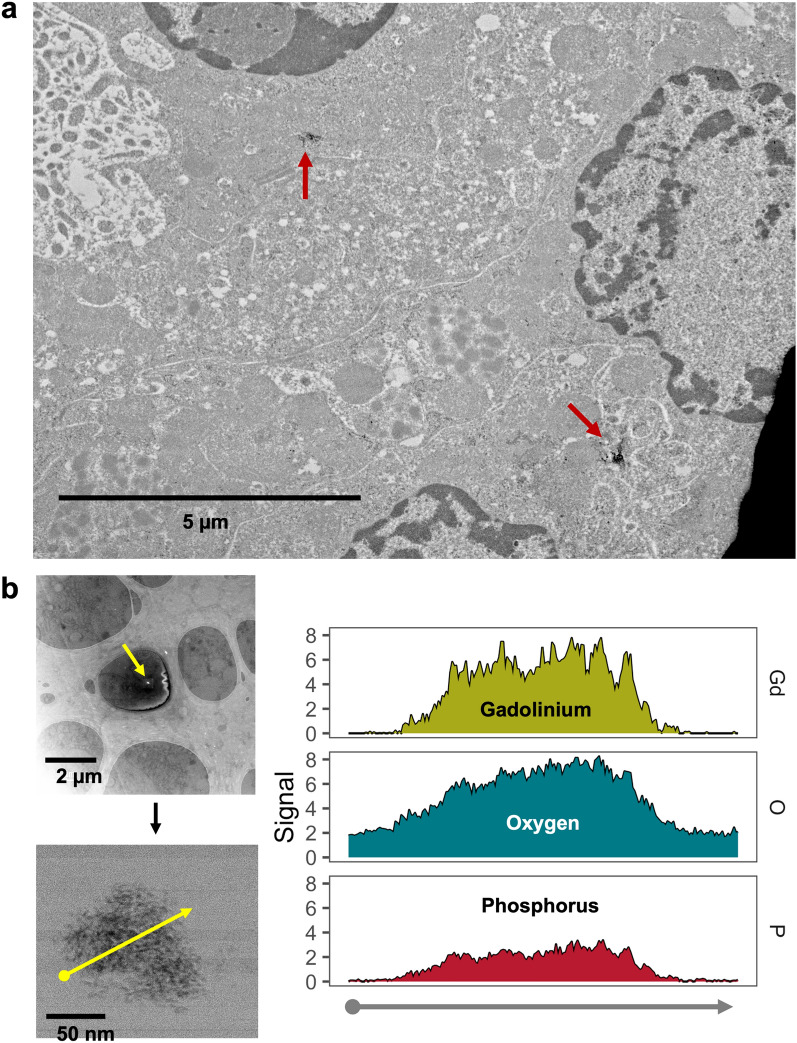
Intracellular gadolinium-rich nanoparticles in human kidneys because of routine diagnostic care. (**a**) Electron-dense nanoparticles in a kidney from a patient with a history of magnetic resonance imaging contrast agent exposure. This kidney was procured 17 days after magnetic resonance imaging contrast agent (20 mL). TEM, Hitachi HT7700. (**b**) The electron-dense nanoparticles are gadolinium rich. Embedded kidney from (**a**) (200 µm sections). XEDS line scanning was performed through an electron-dense nanoparticle. XEDS data revealed gadolinium, oxygen, and phosphorus. JEOL NEOARM 200 kV aberration-corrected scanning transmission electron microscope with dual EDS X-ray analysis system.

**Figure 7:**
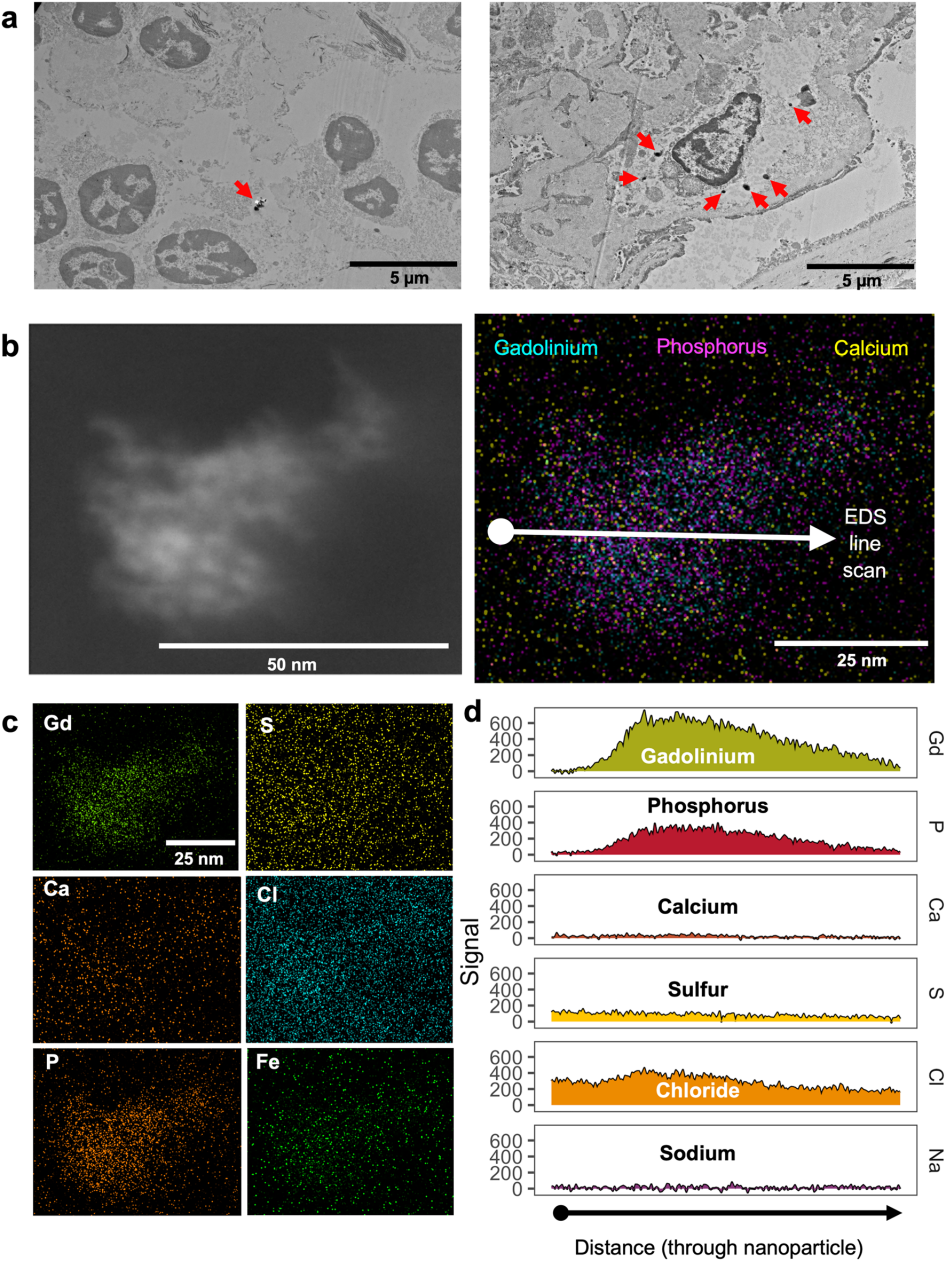
Routine use of MRI contrast agent resulted in intracellular gadolinium-rich nanoparticles. (**a**) Electron-dense intracellular precipitates (arrows) in human kidney. Hitachi H7700 TEM, AMT 16-megapixel digital camera. Calibration bars = 5 µm. (**b**) (Left) Darkfield scanning transmission electron micrograph of an electron-dense precipitate from the kidney specimen depicted in (**a**). (Right) 2D XEDS map of the nanoparticle in (**b**). (**c**) 2D XEDS mapping for gadolinium, calcium, phosphorus, sulfur, chlorine, and iron of the nanoparticle shown in (**b**). (**d**) XEDS line scan through the particle shown in (**b**). JEOL NEOARM 200 kV aberration-corrected scanning transmission electron microscope with dual EDS x-ray analysis system.

**Figure 8:**
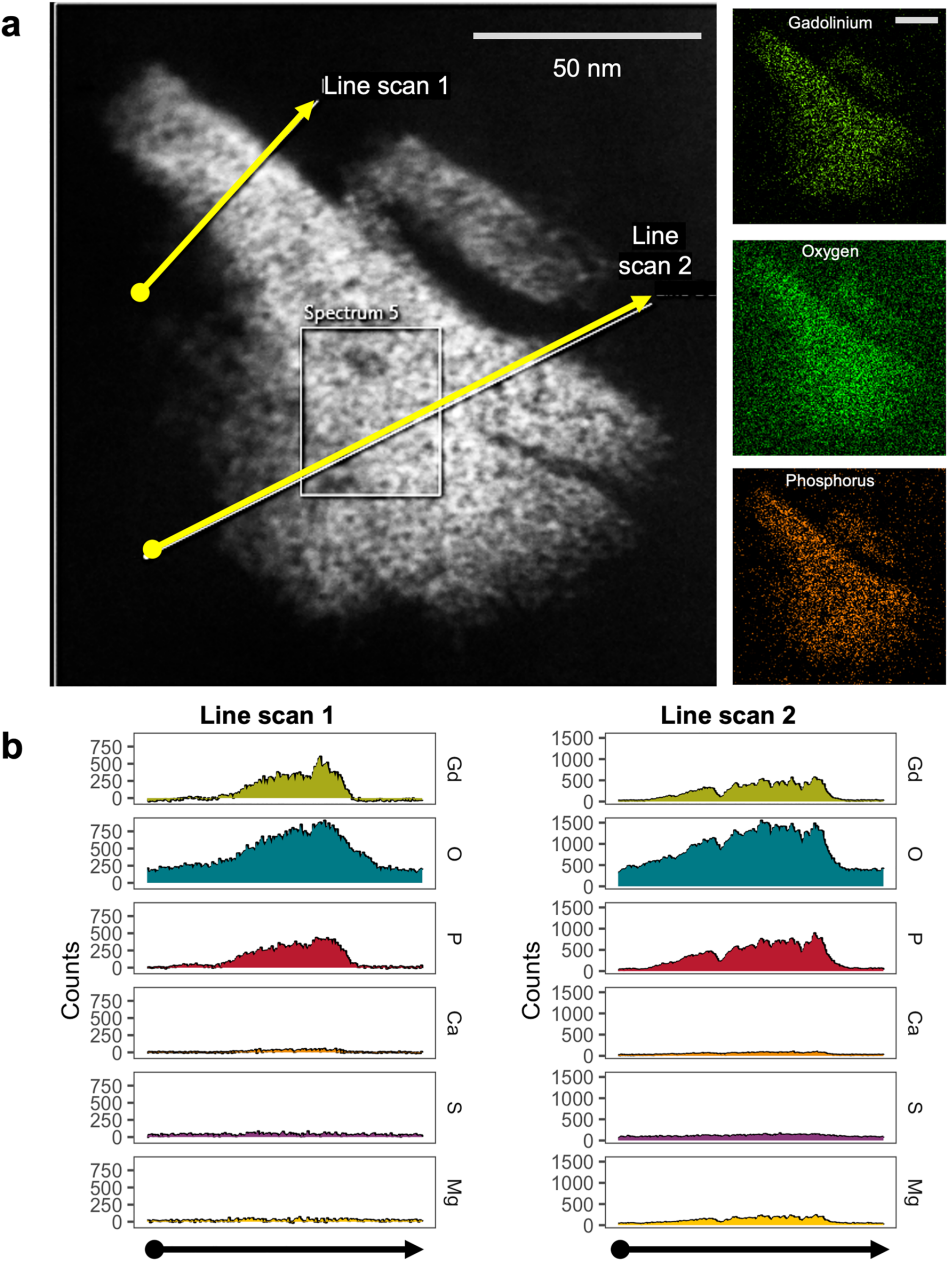
STEM XEDS line scanning through a nanoparticle found in human kidney. (**a**) Dark-field scanning transmission electron microscopy image of a nanoparticle found in human kidney. Insets depict 2D XEDS mapping of the precipitate for gadolinium, oxygen, and phosphorus. Calibration bars = 50 nm. (**b**) XEDS line scan data through the nanoparticle in A showing background corrected X-ray counts as a function of distance along the line scan. JEOL NEOARM (JEM ARM200F), probe aberration-corrected STEM with dual EDS X-ray analysis system.

These human samples were analyzed by TEM and XEDS (University of New Mexico Department of Earth & Planetary Sciences, Fig. 6). Several specimens contained intracellular electron-dense precipitates. The electron densities were roughly 100 nm in diameter. XEDS analysis revealed that these intracellular precipitates contained gadolinium (Fig. 6B). Human tissues were also analyzed by 2D XEDS (Fig. 7). Again, nanoprecipitates showed elevations in gadolinium and phosphorus. STEM XEDS line scans through multiple precipitates (from different patients) again showed a correlation between gadolinium and phosphorus levels (Fig. 8). These results demonstrate that routine MRI contrast agent use leads to lanthanide metallosis.

## Discussion

The affinities of the proprietary chemical formulations of MRI contrast agents for gadolinium do not correlate with the incidences of ‘nephrogenic’/gadolinium-induced systemic fibrosis or gadolinium deposition disease (Supplementary Fig. S14). The amount of time a brand of gadolinium-based contrast agent has been on the market *does* correlate with cases of gadolinium-induced systemic fibrosis and gadolinium deposition disease. Systemic treatment with MRI contrast agents results in the formation of gadolinium-rich nanoparticles in our rodent models^13–15^. Gadolinium-based contrast agent treatment induced various pathological changes in multiple organs of both male and female mice. Herein we provide a detailed atlas of electron microscopic analyses of renal damage from MRI contrast agents with the characterization of gadolinium-rich nanoparticles that form from dechelation and complexation with physiologic elements.

Our findings demonstrate that systemic treatment with MRI contrast agents leads to electron-dense intracellular precipitation within the renal tubular epithelium and interstitial cells in males and females. The formation of spiculated nanoparticles is similar to what has been reported to form from gadolinium oxide (Gd_2_O_3_) in phagolysosomal simulated solutions^24^. (There were no differences in pathology between the sexes.)

Our results also demonstrate gadolinium precipitation in human kidneys as a result of routine MRI contrast agent use. Gadolinium precipitation into an insoluble mineral form demonstrates Le Chatelier’s Principle^25^ in vivo (herein and^13–15^) and in humans*.* The principle of A. L. Le Chatelier and F. Braun is that a chemical equilibrium subject to perturbation (e.g., precipitation of gadolinium) will shift to partially oppose the stress. Because gadolinium precipitates into an insoluble metal-salt form, then the relative affinities of the proprietary pharmaceutical chelates (*log(*_*Ktherm*_*)—*an in vitro measurement^25^) will be perturbed. If gadolinium precipitates out of solution (with phosphate, for example), the equilibrium of this rare earth metal (*Gd*^*3*+^) and the ligand (*L*^*3-*^) will proceed in the following direction,

$$ \begin{aligned} & LGd\left( s \right){ \leftrightharpoons }L^{3 - } \left( s \right) + Gd^{3 + } \left( s \right) \\ & Gd^{3 + } \left( s \right) + PO_{4}^{3 - } \left( s \right) \rightharpoonup GdPO_{4} \left( p \right). \\ \end{aligned} $$

The formation of lanthanide-laden nanoparticles in vivo and the sequellae may be the initial step for the rare earth element metalloses nephrogenic systemic fibrosis and multisymptomatic illnesses such as SAGE. This phenomenon raises important questions regarding the safety of MRI contrast agents.

*Phosphorus in these gadolinium-rich nanoparticles implies these are a type of gadolinium phosphate* (GdPO_4_)*.* Although gadolinium phosphate is not found in nature, it has been detected intracellularly in gadolinium chloride-treated rats^26^.

Delicate biologic specimens are subject to decimation from the high energies of scanning transmission electron microscopes purposed for materials science applications. Herein, we report a method for assessing lanthanide-rich nanostructures in biologic specimens that preserves enough contrast to localize subcellular structures. Our model is similar to that reported in patients with the characteristic renal proximal tubule vacuolization of gadolinium-induced nephropathy^27^.

Rare earth elements, including gadolinium, have unique physical and chemical properties that render them indispensable for critical technologies^28^. Gadolinium usage and indications are rising despite prescribing information boxed warnings of permanent brain retention and sometimes fatal ‘nephrogenic’ systemic fibrosis. The data presented here demonstrate that gadolinium-based contrast agents are not entirely benign. Gadolinium-based contrast agents induce significant pathologic changes in the kidney^13–15,20^ and skin^15–17,19^. Dechelation and precipitation are likely related to the multi-symptom illness reported in patients with gadolinium-induced diseases. Localization, identification, and speciation of retained gadolinium are critical to understanding the mechanisms of toxicity. Our findings are a foundation for understanding the mechanisms of gadolinium-induced disorders and the development of therapies. Rather than dismiss patients who may have suffered from complications due to enhanced MRI procedures, pathologic specimens should be examined for evidence of gadolinium-rich deposits.

Our results suggest that gadolinium is dechelated from MRI contrast agent formulations in vivo and is metabolized into mineralized intracellular nanoparticles. The high concentrations of phosphorus (and oxygen) suggest that the nanoparticles contain insoluble GdPO_4_ (and perhaps Gd_2_O_3_/Gd(OH)_3_) or a more complex/heterogenous mineral. The phosphorus reservoir is unknown. The abundance of phosphorus in lipids and systemic response to gadolinium suggest that leaching from intracellular membranes may be a mechanism. Gadolinium is not a physiologic element. It is reasonable to assume that iatrogenic kidney injury, systemic fibrosis, dermal plaques, and SAGE are all part of a spectrum of disorders resulting from the retention of a toxic lanthanide metal. Nanotoxicity is undoubtedly a mediator of MRI contrast agent complications. Differential decomposition of MRI contrast agents may explain susceptibility to complications.

## Methods

### Animals

All methods were carried out in compliance with relevant guidelines and the study was approved by the University of New Mexico’s Institutional Animal Care and Use Committee (IACUC, protocol 21-201088-HSC, Animal Welfare Assurance # D16-00228, A3350-01, USDA Registration # 85-R-0014). Sex-matched wild-type C57/BL6 mice were randomized by weight into untreated (*n* = 10) or gadolinium-based contrast agent treatment (Omniscan, *n* = *10*) groups^13–18,20,21^. Male C57/BL6 mice weighed 27 g, whereas female C57/BL6 mice weighed 20 g and were 6–8 weeks of age at the start of the experiment. The contrast agent Omniscan was injected intraperitoneally at a dose of 2.5 mmol per kilogram body weight. This dose is equivalent to twice the clinically approved human dose (human equivalent dose) after adjustment for body surface area and is in accordance with the Food and Drug Administration Guidance for Industry^29^. Injections were administered 5 days a week for 4 weeks. The experiments adhered to the ARRIVE guidelines.

### Human pathological specimens

Were obtained from the University of New Mexico Human Tissue Repository (approved by the University of New Mexico Health Sciences Center Institutional Review Board, IRB, protocol #01-313). The experimental protocol was approved by the University of New Mexico Health Sciences Center, Human Research Protections Program/Human Tissue Oversight Committee/Scientific Review Committee (SRC #007-21, de-identified materials, Exempt Category 4 HRP-582; University of New Mexico Health Sciences Center IRB-approved protocol #19-660). All samples were obtained as unidentified in compliance with this protocol. Flash-frozen kidney tissue was obtained from 5 individuals with histories of MRI contrast agent exposure and 5 who were contrast-naïve. The frozen tissue samples with no embedding medium were transported on dry ice from the repository and stored at −80C for further analysis. Pieces (10–15 mg) of frozen tissue were digested and gadolinium concentrations were quantified using PerkinElmer NexION 3000 inductively coupled plasma mass spectrometry with a detection limit of 0.01 ppm. For electron microscopy, flash-frozen tissues were fixed in 3% formaldehyde, 2% glutaraldehyde in phosphate-buffered saline for one hour at room temperature then cut into smaller sections with fresh fixative overnight at 4ºC. Pieces were washed, stained with 1% tannic acid × 1 h, dehydrated, and embedded in epoxy resin. For transmission electron microscopy (TEM), pieces were sectioned at 60–80 nm and placed on copper grids. For darkfield scanning TEM (STEM), pieces were sectioned at 100–200 nm onto holey carbon grids.

### Histology

Tissues were harvested and processed as previously described^14–16,19^. Organs are divided into fixative (10% neutral buffered formalin and electron microscopy as described herein). Kidneys are decapsulated, butterflied, and cortices divided into fixative. Flash-frozen liver tissues were embedded in optimal cutting temperature medium, and cryostat sectioned onto glass slides (70–80 μm) and subsequently stained with lipid dye, oil red O. Microscopy was performed using a Nikon Eclipse E200 microscope coupled with a DS-Fi3 digital camera (Nikon Instruments Inc., Melville, New York). The veterinary pathologist (DK) was blinded to the groups.

### Quantification of hepatic steatosis

Oil red O-stained liver sections were imaged using an oil immersion objective lens (100 ×), and the images digitally analyzed. Images were digitally assessed for lipid area in untreated, and gadolinium-based contrast agent treated livers using Nikon NIS-Elements BR software (Nikon Instruments Inc., Melville, New York).

### Metabolomics

Frozen liver samples were processed by Human Metabolome Technologies (HMT, Japan), and capillary electrophoresis mass spectrometry (CE-MS) was performed. Liver metabolites from gadolinium-treated groups that differed from untreated liver using false discovery rate (FDR, Benjamini and Hochberg method), *P < 0.05, **P < 0.01, ***P < 0.001, were selected for inclusion in this study.

### Electron microscopy

Renal cortices and liver were fixed in glutaraldehyde-containing fixative, post-fixed with 1% tannic acid, embedded in epoxy resin, and sectioned at 200 nm. Semithin sections, without secondary staining, were placed onto carbon holey support grids (Supplementary Fig. S15) for scanning transmission electron microscopy. Conventional transmission electron microscopy was performed on 60–80 nm-thick sections using the Hitachi HT7700 with AMT 16-megapixel digital camera operating at 80 kV. STEM implemented the use of a JEOL 2010F FEGSTEM 200 kV transmission electron microscope (TEM), with Oxford Analytical AZTec X-ray energy-dispersive spectroscopy system, equipped with an XMax 80N 80mm^2^ silicon drift detector (UNM), and the FEI Tecnai G(2) F30 S-Twin 300 kV transmission electron microscope equipped with Fischione Instruments HAADF STEM detectors (CINT). Human kidney sections (200 nm) were mounted on holey carbon grids and scanned with a JEOL NEOARM 200 kV Aberration Corrected scanning transmission microscope (STEM) equipped with two JEOL 100 mm^2^ EDS detectors controlled by Oxford Instruments AZTec software.

### X-ray energy dispersive spectroscopy (XEDS)

Multiple line scan profiles (JEOL 2010F FEGSTEM) were performed on regions of interest. Data were collected for elements of interest; gadolinium (Gd), magnesium (Mg), phosphorus (P), calcium (Ca), sulfur (S), oxygen (O), potassium (K), chlorine (Cl), and silicon (Si). Counts were normalized (indexed) for visualization of XEDS line scan data; the location of the line scan was matched to regions of interest. XEDS analysis was performed using a Tecnai F30 TEM operating at 300 keV with an EDAX XEDS detector. Secondary XEDS analysis of the electron-dense material was performed using an EDAX Octane Elite T Super (70 mm^2^) detector on a monochromated ThermoFisher Scientific Titan transmission electron microscope (300 keV) and the JEOL NEOARM 200 kV Aberration Corrected STEM (described above).

### Statistics

XEDS line scan data for each element were indexed to their total area under the curve. Multiple regression analysis included the index values for elements of comparison, subcellular regions, and sex. Statistical analysis was conducted with RStudio (2022.07.1)/R (version 4.0.3).

## Supplementary Information

Supplementary Information 1.

Supplementary Information 2.

Supplementary Information 3.

Supplementary Information 4.

Supplementary Information 5.

Supplementary Information 6.

Supplementary Information 7.

Supplementary Information 8.

Supplementary Information 9.

Supplementary Information 10.

Supplementary Information 11.

Supplementary Information 12.

Supplementary Information 13.

Supplementary Information 14.

Supplementary Information 15.

Supplementary Information 16.

Supplementary Information 17.

Supplementary Information 18.

## Data Availability

The datasets generated and analyzed during the current study are available in the Kidney Institute of New Mexico repository, (https://digitalrepository.unm.edu/kinm/5/).
