# Supplementary figures and images for: The onset of rare earth metallosis begins with renal gadolinium-rich nanoparticles from magnetic resonance imaging contrast agent exposure
Source: Sci Rep. 2023 Feb 4;13:2025. doi: 10.1038/s41598-023-28666-1 (PMC9899216; doi:10.1038/s41598-023-28666-1)

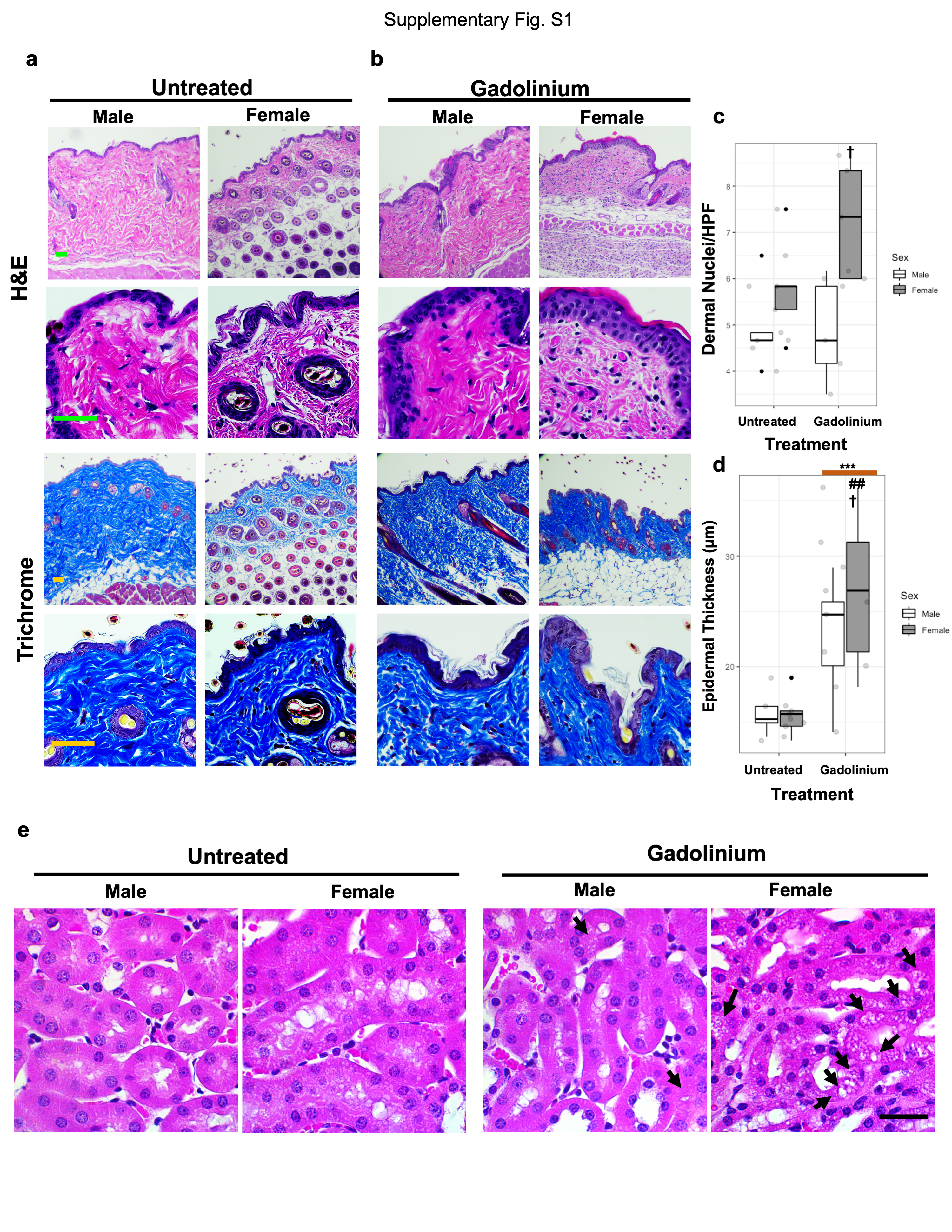

Supplement: Supplementary file 1 — Supplementary Information 1. [file 41598_2023_28666_MOESM1_ESM.png]

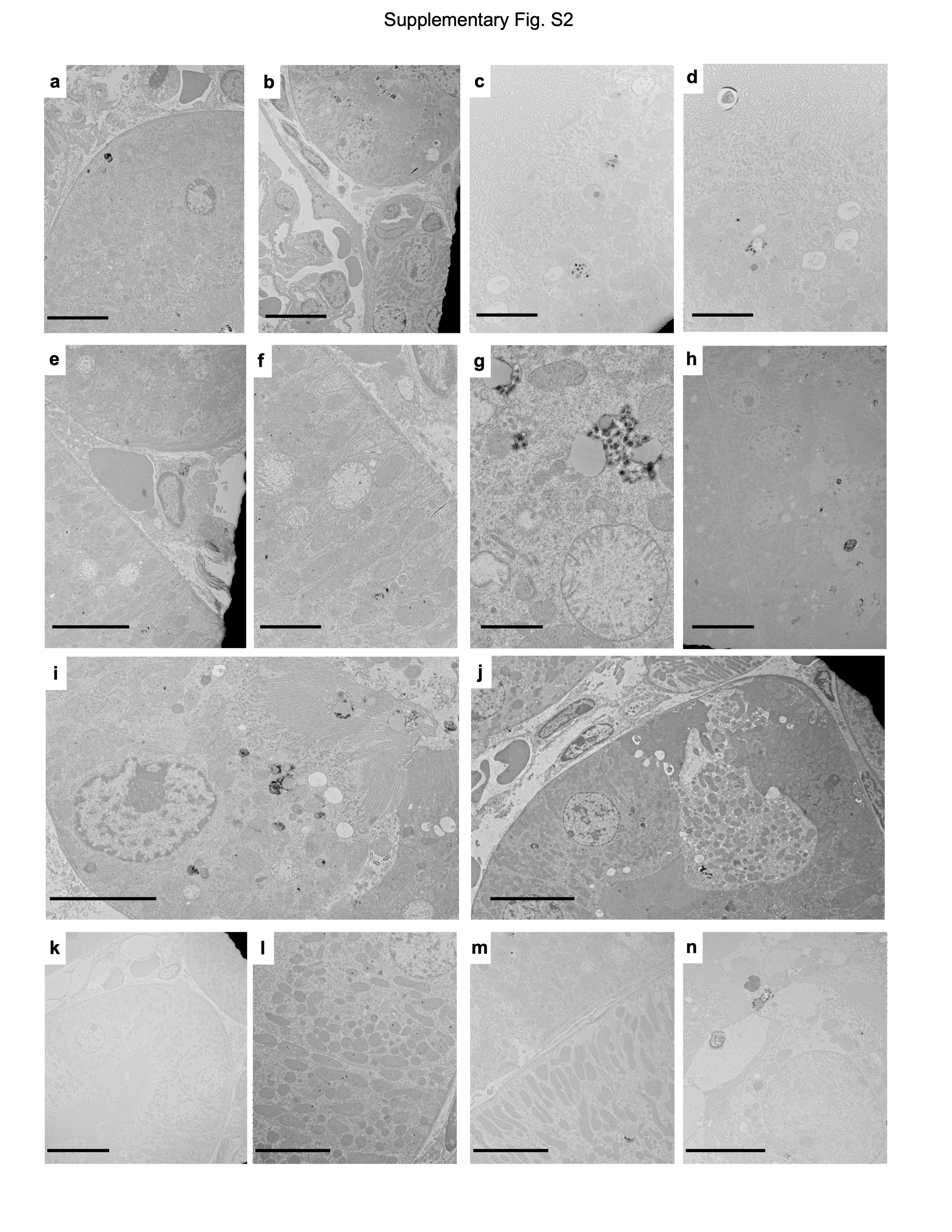

Supplement: Supplementary file 2 — Supplementary Information 2. [file 41598_2023_28666_MOESM2_ESM.png]

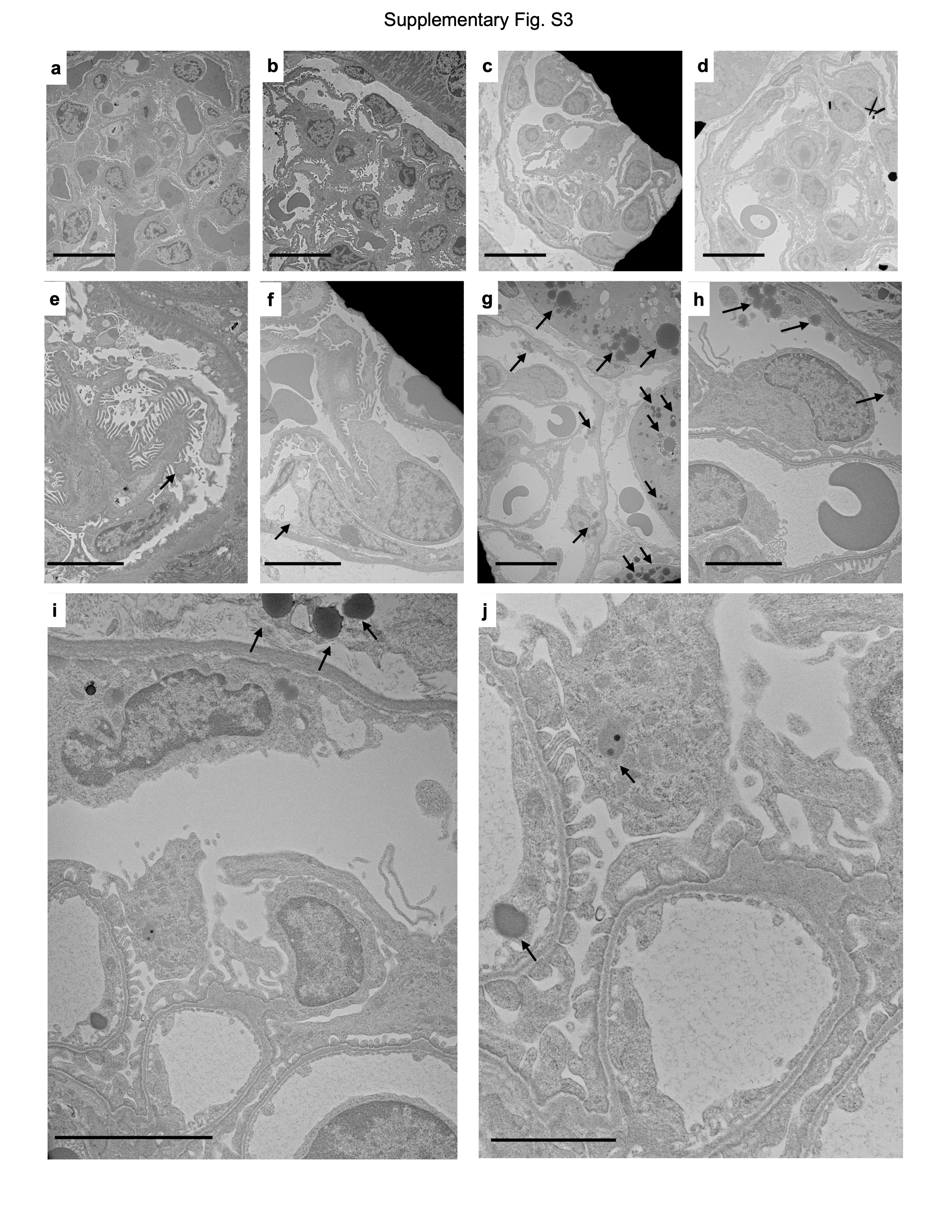

Supplement: Supplementary file 3 — Supplementary Information 3. [file 41598_2023_28666_MOESM3_ESM.png]

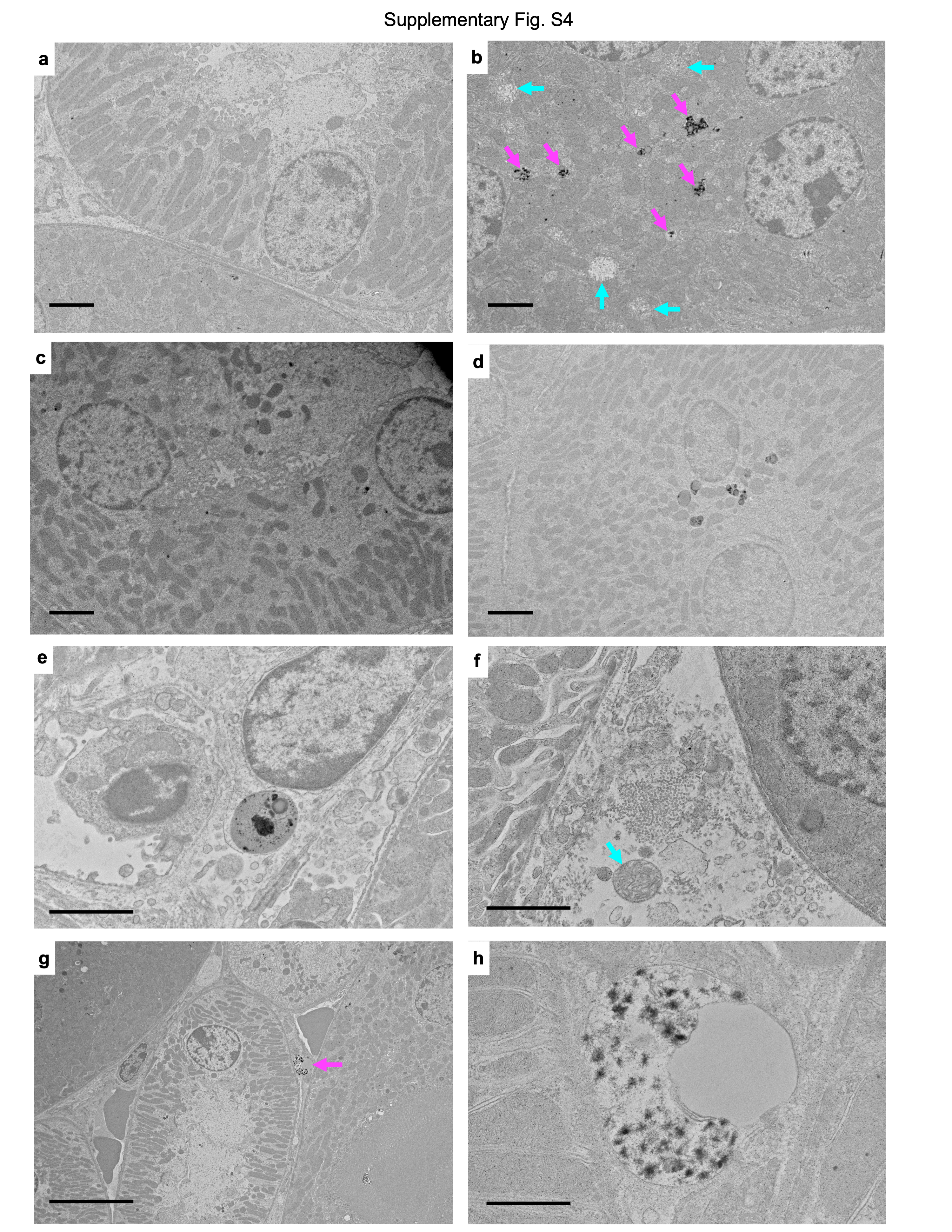

Supplement: Supplementary file 4 — Supplementary Information 4. [file 41598_2023_28666_MOESM4_ESM.png]

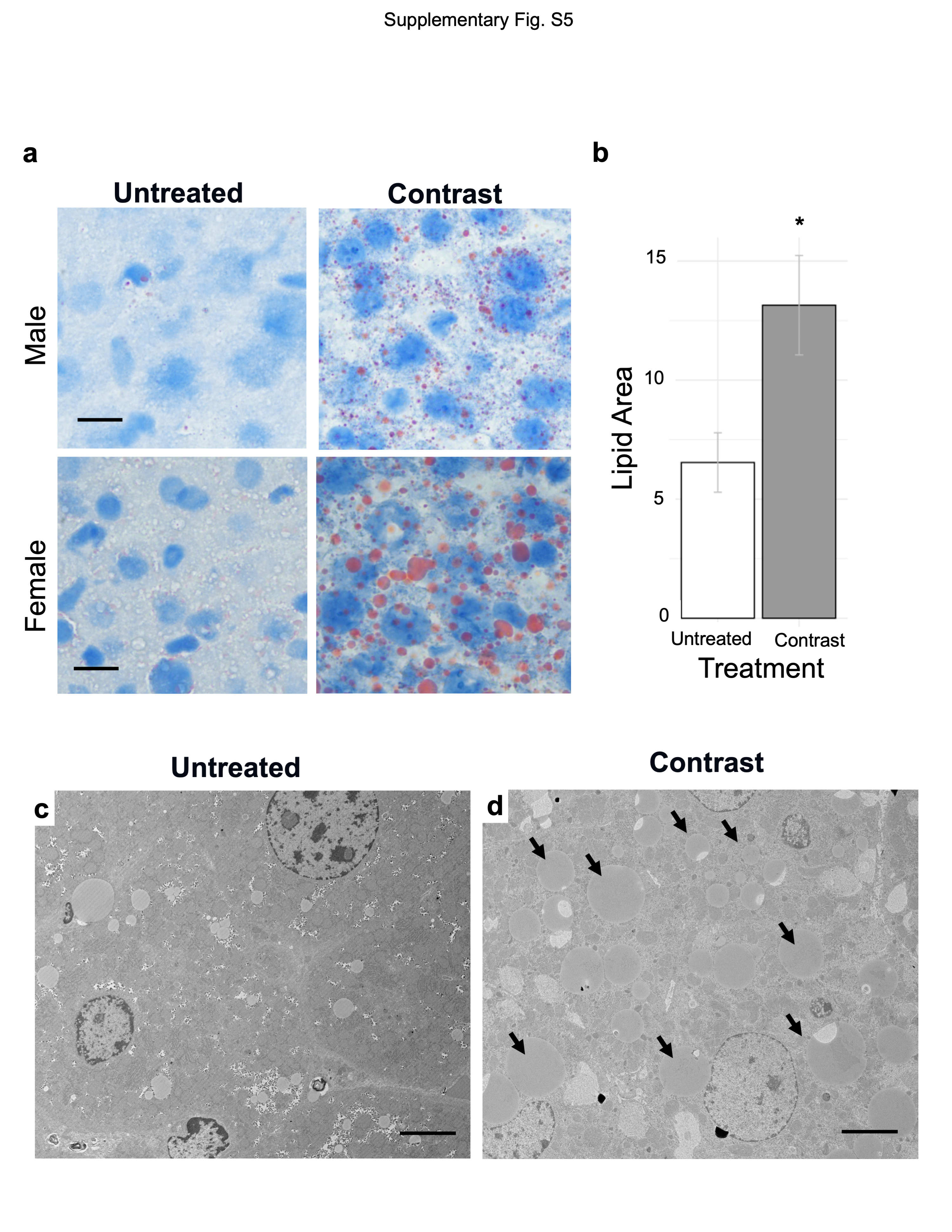

Supplement: Supplementary file 5 — Supplementary Information 5. [file 41598_2023_28666_MOESM5_ESM.png]

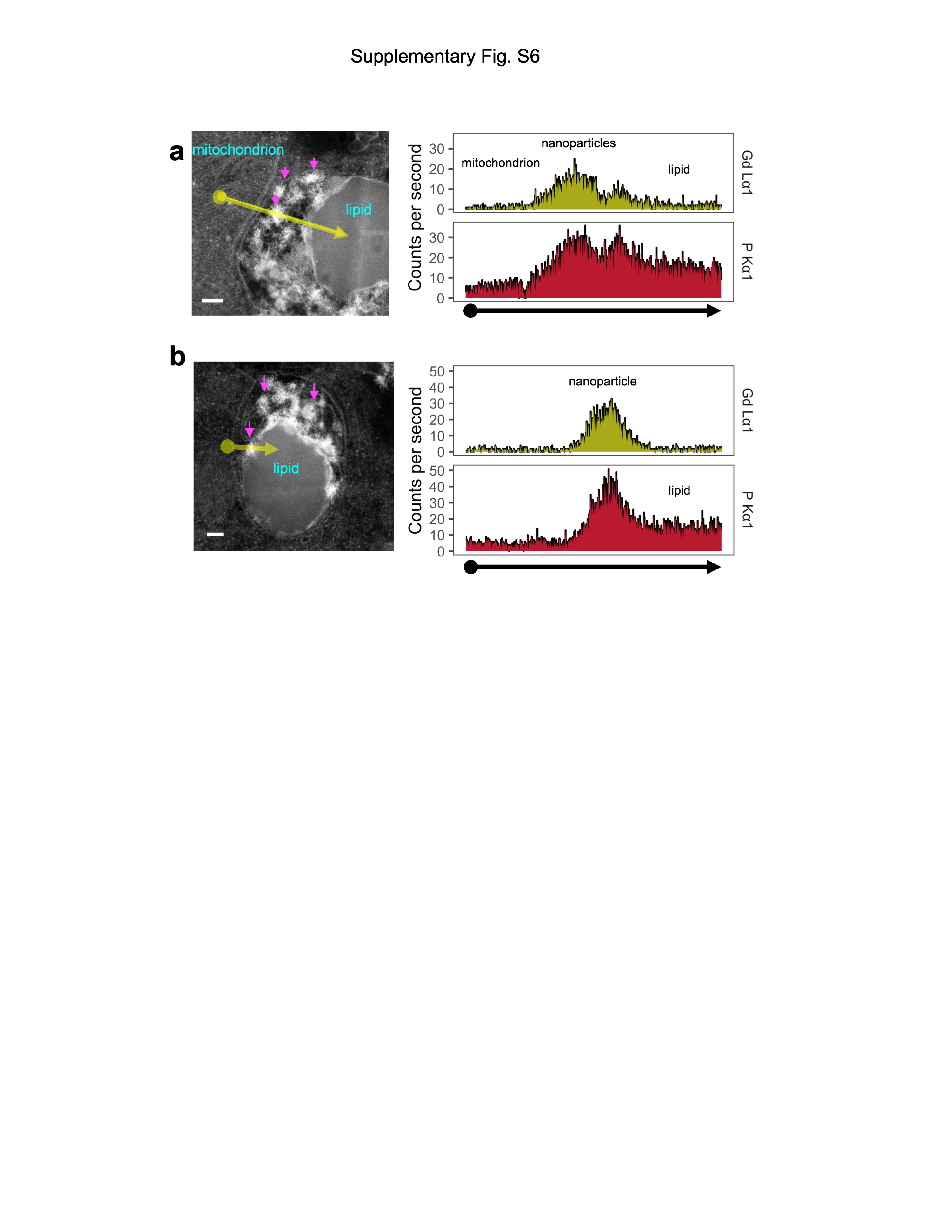

Supplement: Supplementary file 6 — Supplementary Information 6. [file 41598_2023_28666_MOESM6_ESM.png]

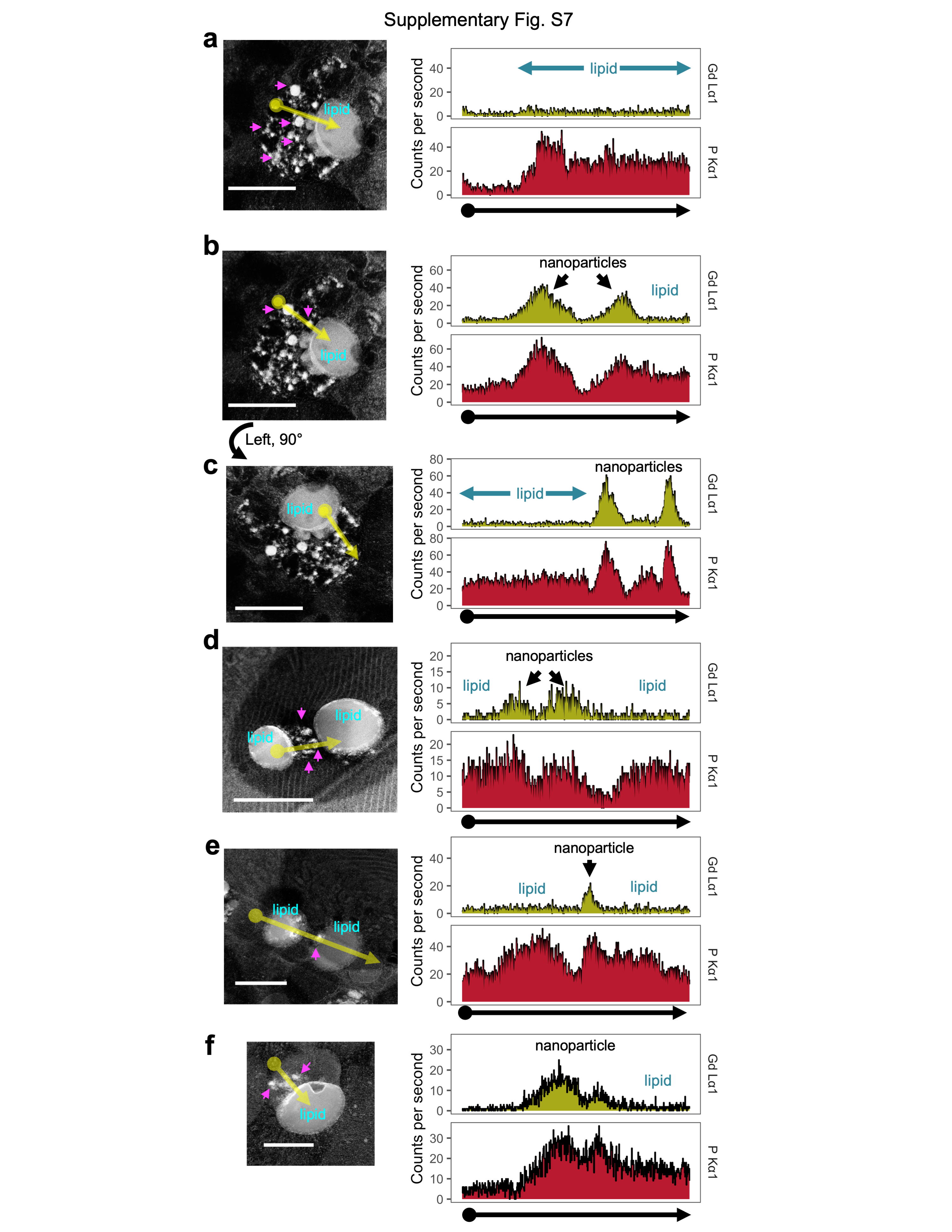

Supplement: Supplementary file 7 — Supplementary Information 7. [file 41598_2023_28666_MOESM7_ESM.png]

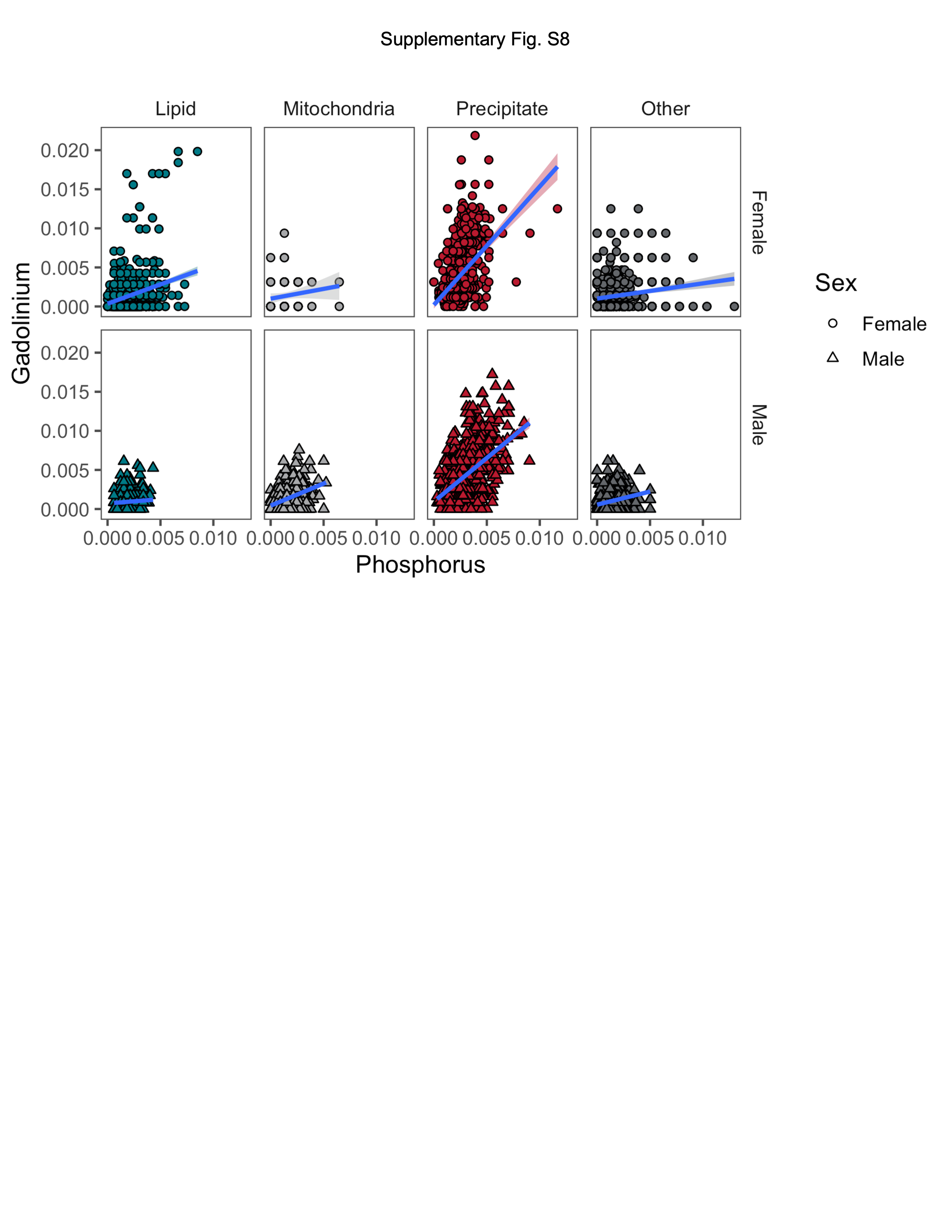

Supplement: Supplementary file 8 — Supplementary Information 8. [file 41598_2023_28666_MOESM8_ESM.png]

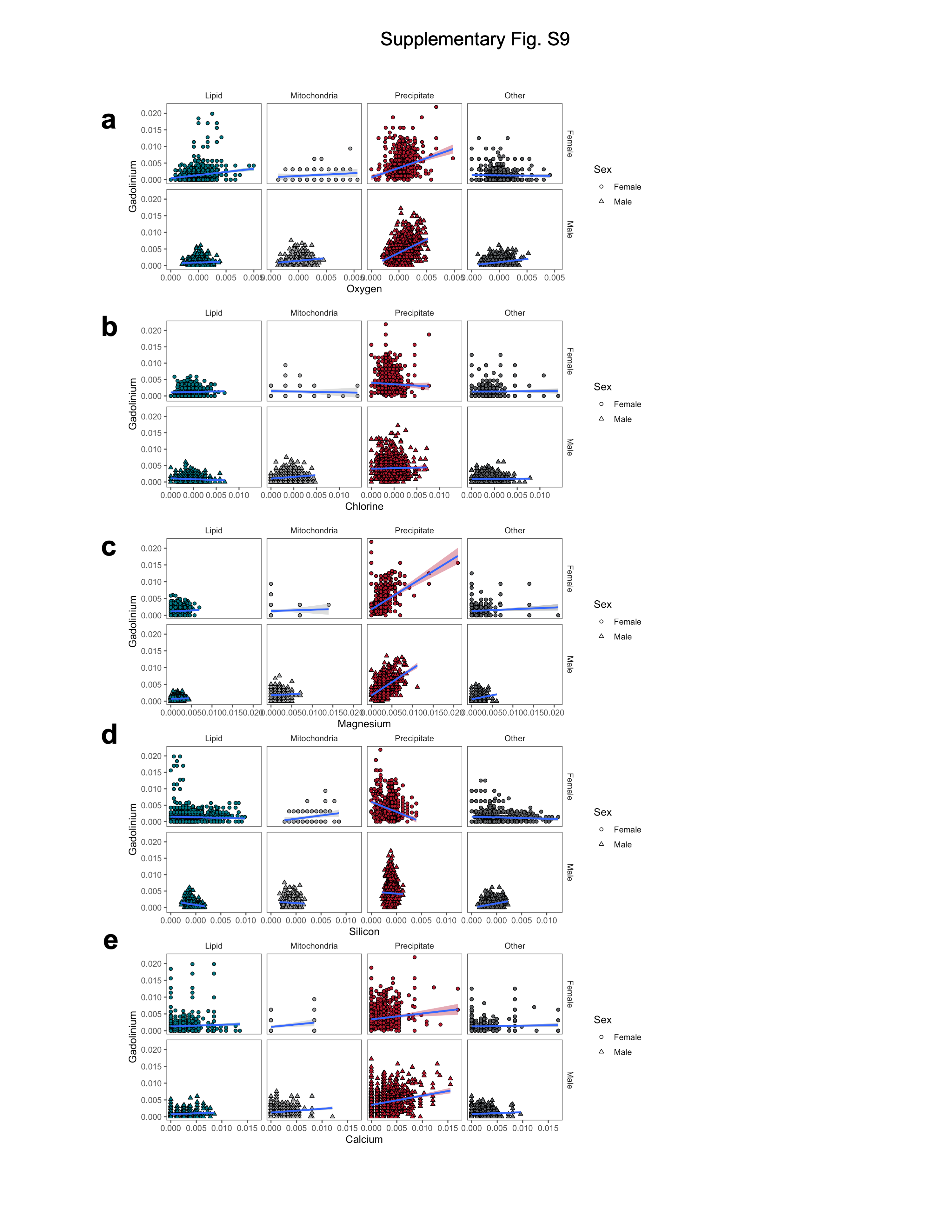

Supplement: Supplementary file 9 — Supplementary Information 9. [file 41598_2023_28666_MOESM9_ESM.png]

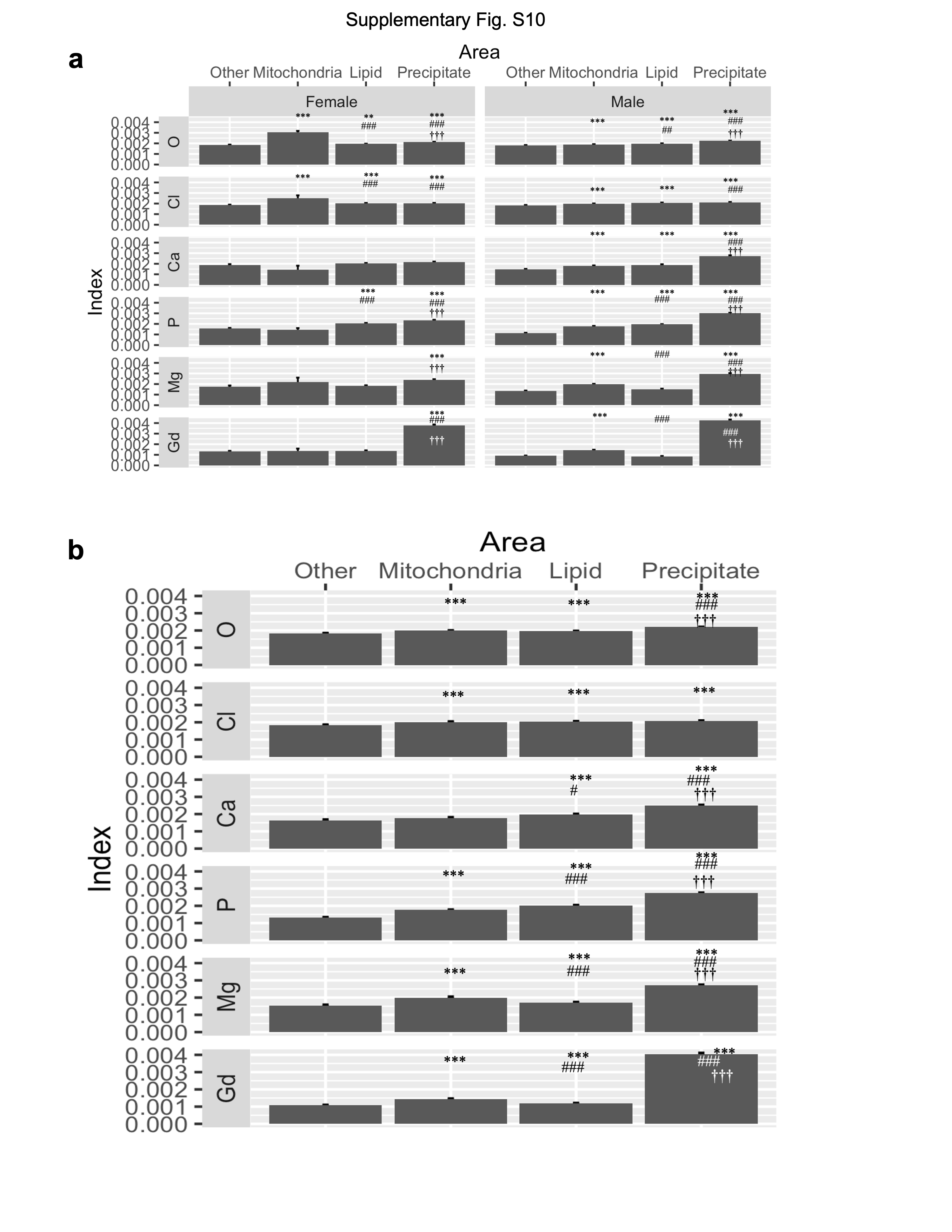

Supplement: Supplementary file 10 — Supplementary Information 10. [file 41598_2023_28666_MOESM10_ESM.png]

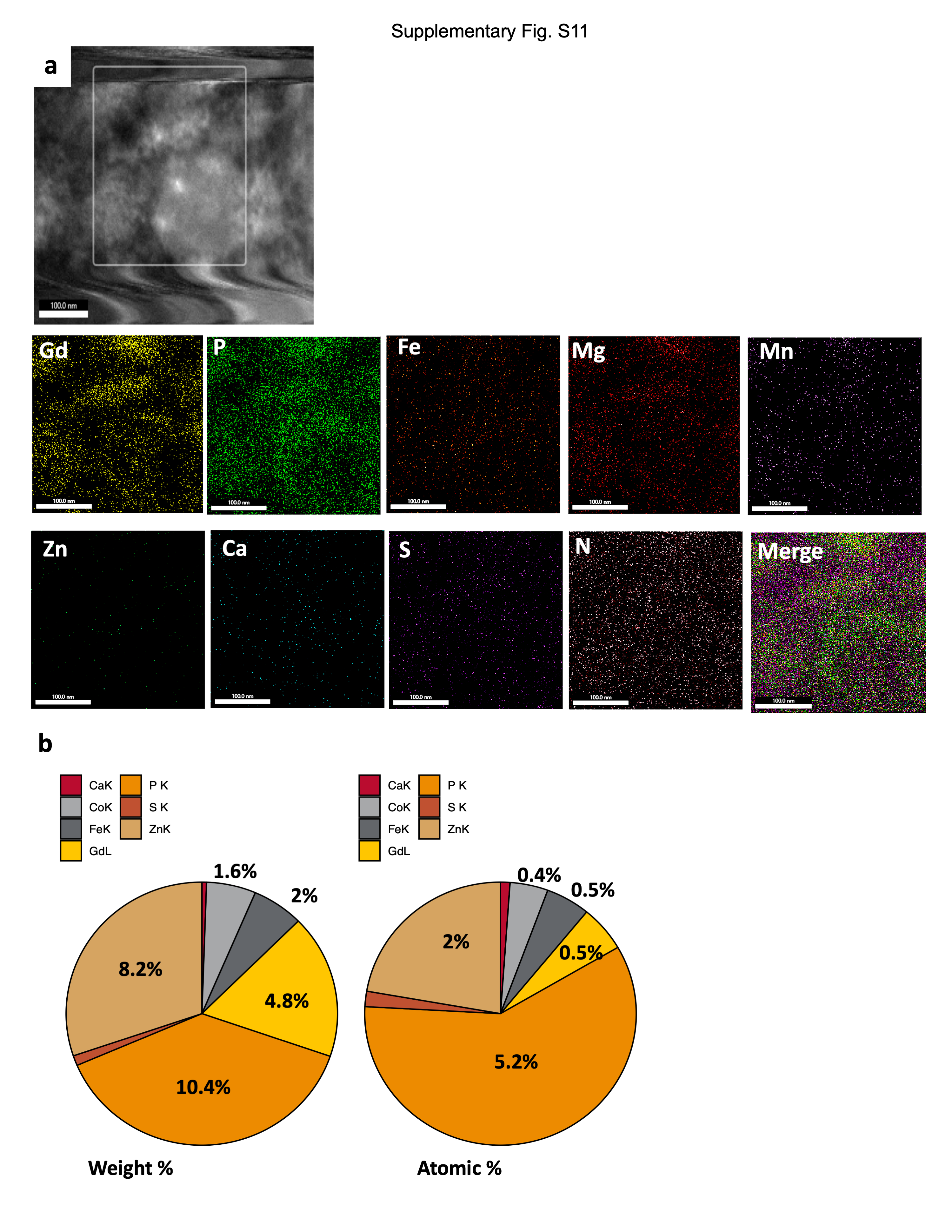

Supplement: Supplementary file 11 — Supplementary Information 11. [file 41598_2023_28666_MOESM11_ESM.png]

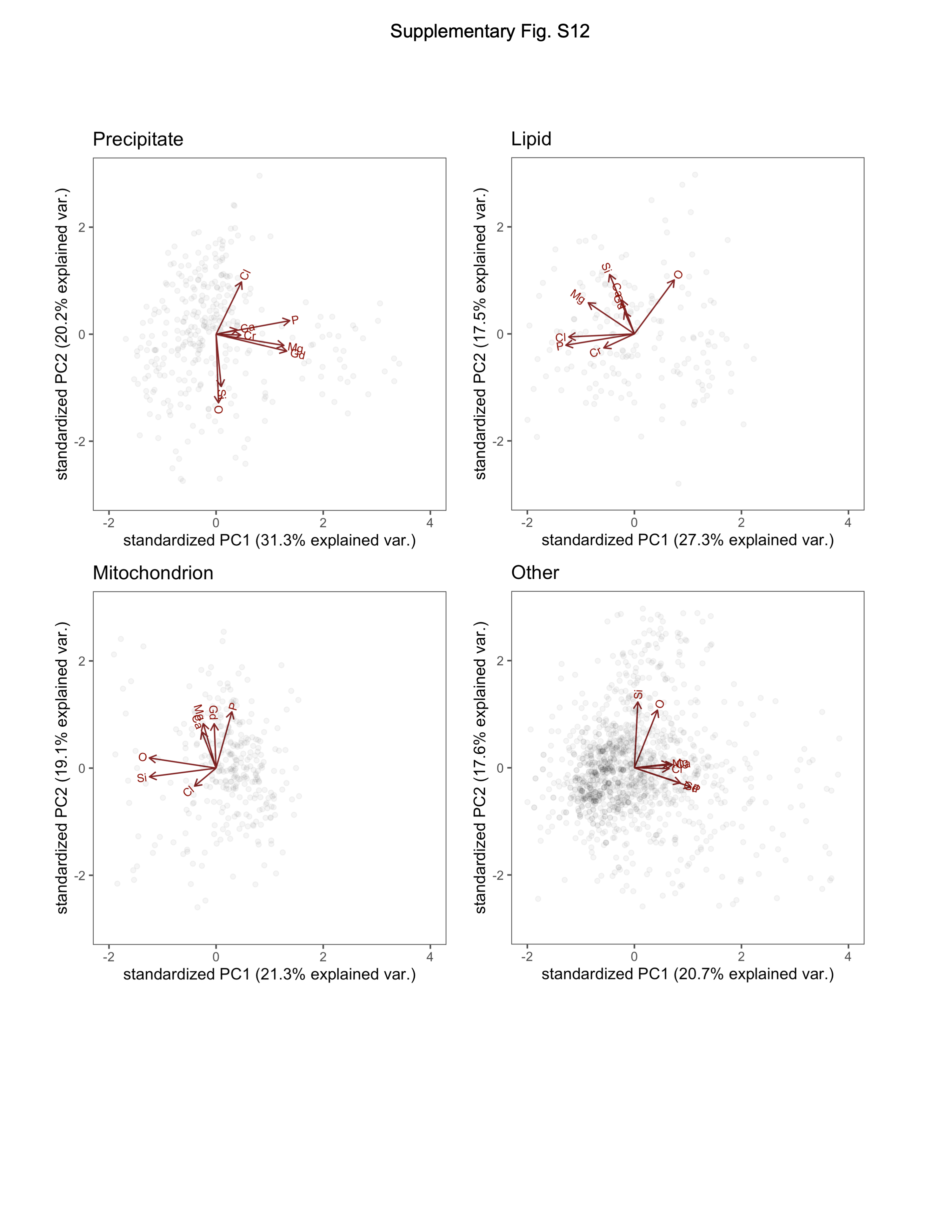

Supplement: Supplementary file 12 — Supplementary Information 12. [file 41598_2023_28666_MOESM12_ESM.png]

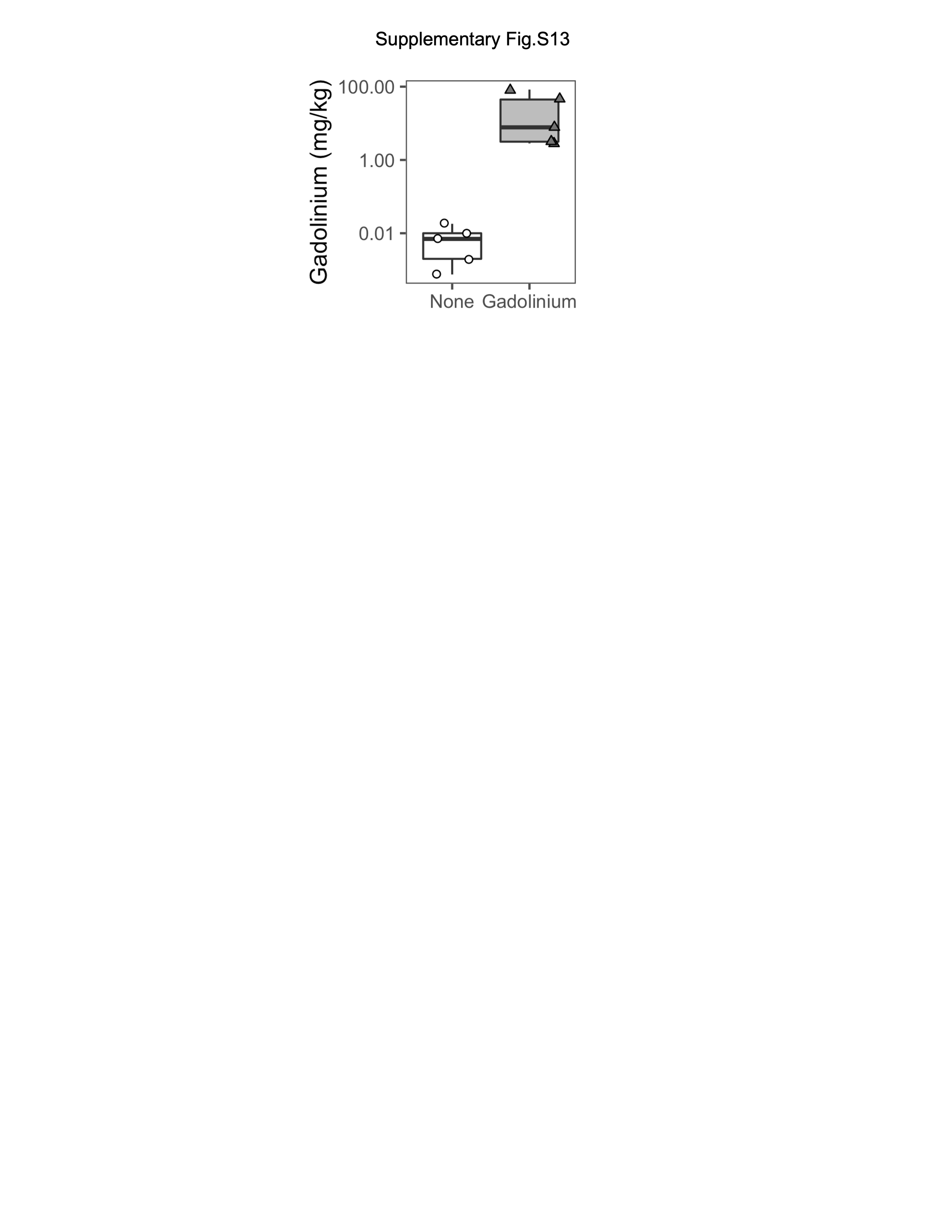

Supplement: Supplementary file 13 — Supplementary Information 13. [file 41598_2023_28666_MOESM13_ESM.png]

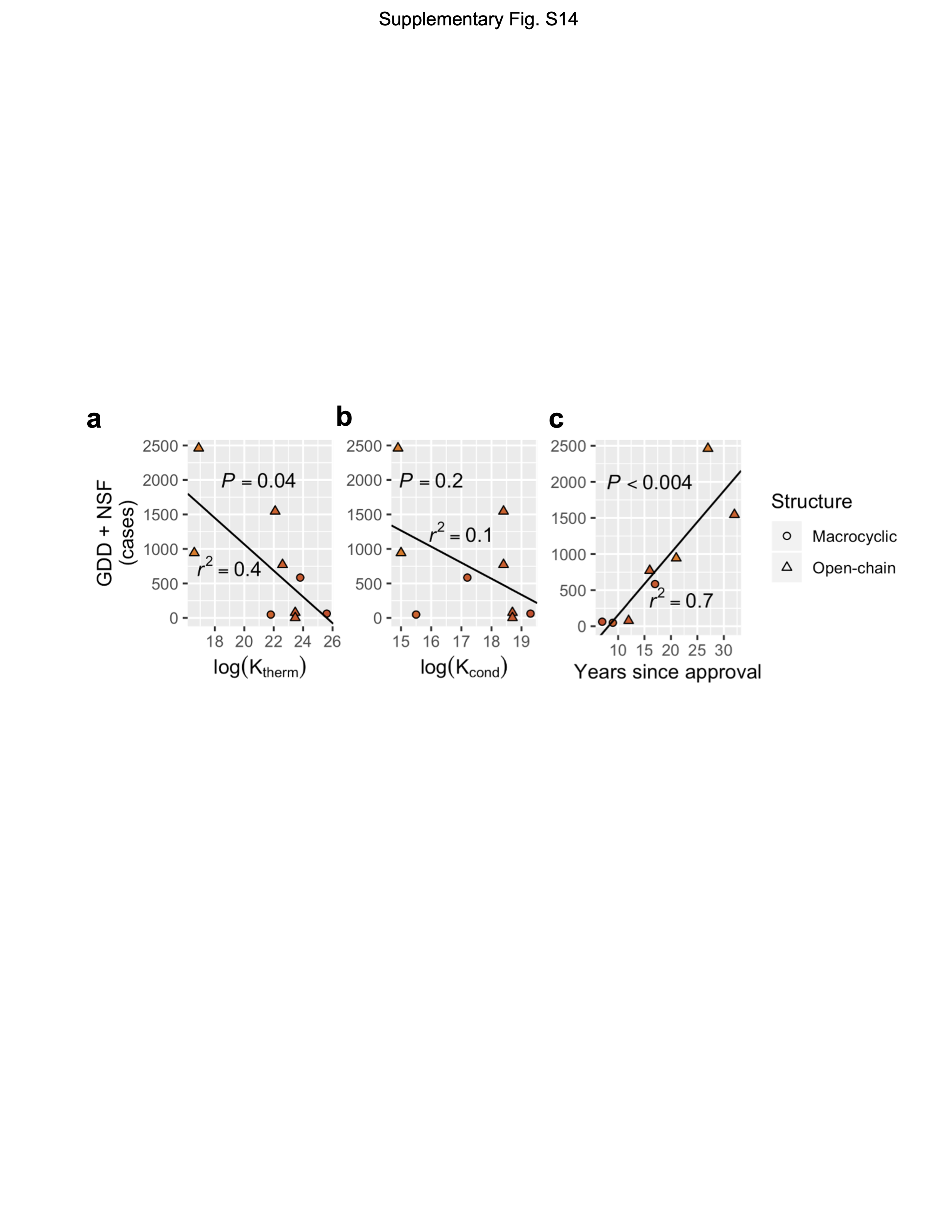

Supplement: Supplementary file 14 — Supplementary Information 14. [file 41598_2023_28666_MOESM14_ESM.png]

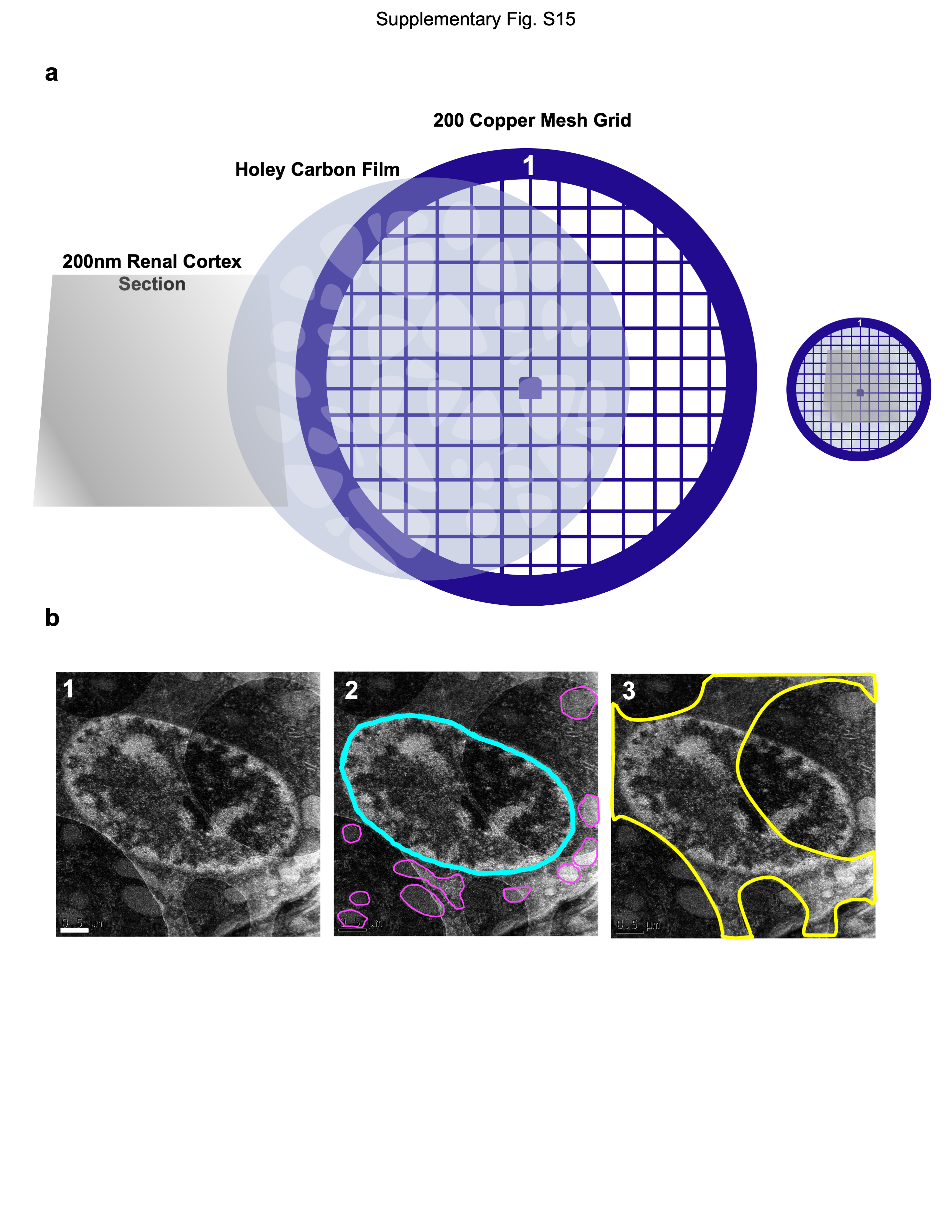

Supplement: Supplementary file 15 — Supplementary Information 15. [file 41598_2023_28666_MOESM15_ESM.png]
