# Supplementary material for: The onset of rare earth metallosis begins with renal gadolinium-rich nanoparticles from magnetic resonance imaging contrast agent exposure
Source: Sci Rep. 2023 Feb 4;13:2025. doi: 10.1038/s41598-023-28666-1 (PMC9899216; doi:10.1038/s41598-023-28666-1)
Supplement: Supplementary file 16 — Supplementary Information 16. [file 41598_2023_28666_MOESM16_ESM.docx]

**Supplementary Fig. S1. Characterization of magnetic resonance imaging (MRI) contrast agent-induced epidermal changes.** **a,** Representative lumbar skin hematoxylin & eosin (H&E) and trichrome-stained sections from untreated male and female mice. Calibration bar = 50 µm. **b,** H&E and trichrome images demonstrating gadolinium-induced pathologic changes in male and female treated mice. Calibration bar = 50 µm. **c,** Quantification of nuclei in multiple dermal regions of interest (ROI). †*P* < 0.01 from third lane. **d,** Analysis of epidermal thickness from untreated and MRI contrast agent-treated groups. ****P* < 0.001 from first and second lanes (untreated), ##*P* = 0.01 from second lane, †*P* < 0.05 from third lane. **e,** MRI contrast agent-induced vacuolation in renal proximal tubular cells. Compared to the control groups, the renal cortex from MRI contrast agent-treated mice demonstrated vacuolization (arrows). Proximal tubular vacuolization was observed in gadolinium-treated mice, as previously reported. This intracytoplasmic vacuolization is distinct from the foamy appearance of material within tubule lumina. Hematoxylin & eosin (H&E), 60X. Calibration bar = 30.0 μm. Nikon Eclipse E200 microscope, DS-Fi3 digital camera. ​

**Supplementary Fig. S2.** **Transmission electron micrographs of MRI contrast agent-treated male mice demonstrated multiple renal proximal tubular abnormalities.** **a,** Proximal tubular segment from an untreated male mouse. Calibration bar = 5 µm. **b,** Proximal tubule and glomerulus from a MRI contrast agent-treated male. Proximal tubules demonstrated an increase in cytoplasmic vesicles, often containing electron-dense material. Calibration bar = 10 µm. **c,** Apical cytoplasmic bodies in a proximal tubule cell with electron-dense, spiculated precipitates from a MRI contrast agent-treated male and swollen mitochondria. Calibration bar = 2.0 µm. **d,** Large apical cytoplasmic vesicles, one with spiculated electron-dense material, in the renal proximal tubule from a MRI contrast agent-treated male. Calibration bar = 2 µm. **e,** Dysmorphic mitochondria (arrows), often with decreased cristae and increased matrix, neighboring mitochondria displaying shortened structure, in renal proximal tubules from a MRI contrast agent-treated male. Calibration bar = 5 µm. **f,** Magnification of basolateral proximal tubular cell in (e). Calibration bar = 2.0 µm. **g,** Swelling of mitochondria and cytoplasmic vesicles with electron-dense, spiculated material in a renal proximal tubule from a MRI contrast agent-treated male. Calibration bar = 1.0 µm. **h,** Ruptured apical surface of a renal proximal tubular cell in a MRI contrast agent-treated male. Calibration bar = 10 µm. **i,** Apical cytoplasmic blebbing in renal proximal tubules. Cytoplasmic vacuoles were increased, often lipid-laden and with electron densities. Toxic mitochondria were also prevalent. Calibration bar = 5.0 µm. **j,** Necrotic renal proximal tubular cell with interstitial expansion and increased interstitial cellularity. Cytoplasmic vacuoles were enlarged and numerous. Calibration bar = 10 µm. **k,** Renal proximal tubule from a MRI contrast agent-treated male demonstrating reduction of mitochondrial density. There is a concomitant expansion of the interstitium. Calibration bar = 10 µm. **l,** Magnification of the area in (k). Calibration bar = 2 µm. **m,** Ruptured renal proximal tubular cell basement membrane (arrow) from a MRI contrast agent-treated male. Mitochondriopathy and lipid-laden vacuoles with electron-dense material were also present. Calibration bar = 5 µm. **n,** Ruptured apical membrane of a renal proximal tubular cell (with many cytoplasmic bodies, some with electron-dense material) from a MRI contrast agent-treated male. Calibration bar = 5.0 µm. Hitachi H7700 TEM, AMT 16-megapixel digital camera.

**Supplementary Fig. S3. The effects of MRI contrast agent treatment on renal glomeruli.** **a–d,** Renal glomeruli from untreated male (a) and female mice (b) and from MRI contrast agent-treated male (c) and female mice (d). Calibration bars = 10 µm. **e–f,** Parietal cells from MRI contrast agent-treated male (e) and MRI contrast agent-treated female (f) mice demonstrated vacuolization (arrows). Calibration bar = 5 µm. **g,** Renal glomerular parietal cells from a MRI contrast agent-treated female demonstrating unilamellar (lipid) intracellular inclusions. Calibration bar = 10 µm. **h,** Higher magnification of region in (g), with lipid-laden vacuoles (arrows) similar to those seen in proximal tubular cells of MRI contrast agent-treated mice. Calibration bar = 5 µm. **i,** Ruptured podocyte and renal glomerular endothelial cell and glomerular parietal cell with electron-dense intracellular inclusions. Calibration bar = 5 µm. **j,** Magnified region from (i). Calibration bar = 2 µm. Hitachi H7700 TEM, AMT 16-megapixel digital camera.​

**Supplementary Fig. S4. Magnetic resonance contrast agent-induced alterations in renal tubular epithelium.** **a,** Renal distal convoluted tubule (DCT) from untreated male, longitudinal mitochondrial axes perpendicular to basement membrane. Calibration bar = 2 µm. **b,** Electron-dense material (magenta arrows) and dysmorphic mitochondria (cyan arrows). Calibration bar = 2 µm. **c,** Renal distal tubular epithelia from an untreated female. Calibration bar = 2 μm. **d,** Lipid-laden vacuoles with electron-dense material in the renal distal tubular epithelia of a MRI contrast agent-treated female. Calibration bar = 2 µm. **e,** Interstitial area adjacent to damaged proximal tubule, perinuclear intracellular inclusion in kidney from a MRI contrast agent-treated mouse. Calibration bar = 2 µm. **f,** Interstitial expansion with collagen deposition in MRI contrast agent-treated male. A singular mitochondrion—acellular (cyan arrow)—is present in the interstitial space. Calibration bar = 2 µm. **g,** Lipid droplet/unilamellar vesicle and electron-dense material in the renal interstitium of a contrast-treated male. Calibration bar = 10 µm. **h,** Magnified interstitial region from (g). The cytoplasmic vesicle contains electron-dense nanoparticles in the vicinity of a large unilamellar vesicle/lipid droplet. Calibration bar = 1 µm. Hitachi H7700 TEM, AMT 16-megapixel digital camera.

**Supplementary Fig. S5. Magnetic resonance imaging contrast agent-induced changes in the liver.** **a,** Representative images of oil red O-stained livers demonstrated increased triglyceride staining in contrast-treated male and female mice. Calibration bar = 10μm. **b,** The lipid area was increased in contrast-treated animals (*n* = 4 each for each group); **P* < 0.05 by two-tailed t-test. **c,** Transmission electron micrograph of liver from the untreated group. **d,** Magnetic resonance contrast agent treatment induced ballooned hepatocytes, atypical nuclei, increased lipid accumulation (arrows), and reduced mitochondrial volumes. Nikon Eclipse E200 microscope, DS-Fi3 digital camera. Hitachi H7700 TEM, AMT 16-megapixel digital camera.​

**Supplementary Fig. S6.** **X-ray energy-dispersive spectroscopic (XEDS) scanning transmission electron microscope line scan profiles of subcellular regions in gadolinium-treated male proximal tubules.** **a,** Dark-filed scanning transmission electron micrograph (STEM) showing XEDS line scan (yellow arrow) through mitochondrion, nanoparticles (magenta arrows), and unilamellar (lipid) body. Calibration bar = 200 nm. Corresponding XEDS linescan data (X-ray intensity in counts per second vs. distance) for elements of interest, gadolinium (Gd), and phosphorus (P) in regions denoted in STEM micrograph. **b,** XEDS line scan through nanoparticle (magenta arrows), and unilamellar (lipid) inclusion. The corresponding XEDS line scan data demonstrates high gadolinium and phosphorus content in the nanoparticle. The unilamellar bodies contain higher phosphorus than mitochondria or cytoplasm. Calibration bar = 100nm. JEOL 2010F FEGSTEM 200 kV scanning transmission electron microscope, with Oxford Analytical AZTec XEDS system, equipped with XMax 80N 80mm^2^ silicon drift detector.

**Supplementary Fig. S7.** **X-ray energy-dispersive spectroscopic (XEDS) scanning transmission electron microscope line scans through subcellular regions of in proximal tubule cells of magnetic resonance imaging contrast agent-treated female mice.** **a,** Dark-field scanning transmission electron micrograph depicting STEM EDS line scan through region devoid of electron densities into lipid (bypassing electron-dense precipitates). Calibration bar = 2.5 μm. Spectral intensities of gadolinium (Gd L_α1_ region) and phosphorus (P K_α1_ region) are shown as a function of distance along the line profile. **b,** EDS line scanning through the same region as shown in (a) crossing two nanoparticles (magenta arrows) into a lipid droplet. Calibration bar = 2.5 μm. There are two peaks for gadolinium (Gd) corresponding to the nanoparticles in the STEM micrograph. **c,** Photograph from (b) is rotated to correspond with the STEM EDS line scan from inside the lipid droplet through two precipitates within a vacuole. Double-peak profile for both Gd and P from the corresponding nanoparticles, showing colocalization. Calibration bar = 2.5 μm. **d,** EDS line scan spanning two lipid droplets and nanoparticles (magenta arrows) and corresponding XEDS line scan data profile. Calibration bar = 5 μm. **e,** STEM EDS line scan passing through lipids and electron-dense material at lipid interface (arrowhead). High Gd intensity peak corresponds with electron-dense material. Calibration bar = 5 μm. **f,** STEM EDS line scan passing from non-lipid region through nanoparticles (magenta arrows) and lipid with electron-dense border. Line scan data from nanoparticles and lipid border demonstrate colocalizing gadolinium and phosphorus peaks. Calibration bar = 2.5 μm.

**Supplementary Fig. S8.** **Correlation between gadolinium and phosphorus quantities in subcellular regions (unilamellar vesicles/lipid droplets, mitochondria, electron-dense precipitates, and other).** XEDS line scan data through subcellular regions were obtained from MRI contrast agent-treated males (*n* = 3) and females (*n* = 3). Signal intensities were indexed for total area under the curve and fit to a linear model. Linear modeling (ordinary least squares) of gadolinium and phosphorus quantities was significant for unilamellar vesicles/lipid bodies (***r^2^*** = 0.07 in females), mitochondria (***r^2^*** = 0.15 in males), and precipitates (***r^2^*** = 0.22 in females, ***r^2^*** = 0.25 in males).

**Supplementary Fig. S9.** **Regression analysis of gadolinium and elements of interest in proximal tubule intracellular regions.** XEDS line scan data were indexed to the total areas under the curve for each element, then categorized by subcellular region. **a,** Relationship between gadolinium (Gd) and oxygen in regions of interest in female and male gadolinium-treated animals. (***r^2^*** = 0.02, 0,01, 0.1, and 0.003 for lipid, mitochondria, precipitate and other, respectively). **b,** Correlation of Gd and chlorine in intracellular regions and electron-dense precipitates in female and male treated mice. (*B* = 0.0002, 0.007, 2 × 10^-6^, and 0.000 for lipid, mitochondria, precipitate and other, respectively). **c,** Gadolinium and magnesium content in the electron-dense precipitates of treated groups. (***r^2^*** = 0.008, 0.003, 0.2, and 0.03 for lipid, mitochondria, precipitate and other, respectively). **d,** Association of Gd and silicon in lipid, mitochondria, electron-dense nanoparticles, and other background intracellular regions of treated animals. **e,** Calcium and Gd relationship in gadolinium-induced nanoparticles in female and male treated mice. (***r^2^*** = 0.005, 0.03, 0.03, and 0.002 for lipid, mitochondria, precipitate and other, respectively).

**Supplementary Fig. S10**. **Indexed spectral counts for subcellular regions assayed by XEDS line scanning. a,** Spectral counts indexed for quantitation of elements of interest including oxygen (O), chlorine (Cl), calcium (Ca), phosphorous (P), magnesium (Mg), and gadolinium (Gd) in treated female and male groups. **b,** Sum of indexed spectral counts for all line scans from treated groups (both sexes). ****P* < 0.001 from first lane; #*P* < 0.05, ###*P* < 0.001, from second lane; †††*P* < 0.001 from third lane by analysis-of-variance and Tukey honestly significant difference post-hoc testing. JEOL 2010F FEGSTEM 200 kV scanning transmission electron microscope, with Oxford Analytical AZtec XEDS system, equipped with XMax 80N 80mm^2^ silicon drift detector.

**Supplementary Fig. S11.** **Electron-dense precipitates are rich in gadolinium, phosphorus, and other metals.** **a,** Dark-field STEM of a nanoparticle (Top) and the 2-dimensional (2D) XEDS maps for gadolinium (Gd), phosphorus (P), iron (Fe), magnesium (Mg), manganese (Mn), zinc (Zn), calcium (Ca), sulfur (S), and nitrogen (N). Calibration bar = 100nm. **b,** Weight and atomic percentages of elements present in the gadolinium-induced electron-dense precipitates. *n* = 2 individual precipitates. ThermoFisher Scientific Titan transmission electron microscope (300keV), with an Octane Elite T Super (70mm^2^) detector.

**Supplementary Fig. S12**. **Principal component analyses of indexed spectral signals for precipitates, lipid bodies, mitochondria, and other intracellular areas.** Many elements were measured (Ca, Cl, Cr, Gd, P, O, Mg, Si,) in four intracellular categories (nanoparticulate precipitates, unilamellar/lipid bodies, mitochondria, and other/cytosolic regions). Elements were used as parameters. Gadolinium and phosphorus continued to possess the largest principal components in the nanoparticles, 0.55 and 0.57, respectively.

**Supplementary Fig. S13. Gadolinium is detectable in kidneys from humans with histories of magnetic resonance imaging contrast agent exposure**. Kidney tissues were obtained from gadolinium-naïve (*n* = 5) and magnetic resonance imaging contrast agent exposed individuals (*n* = 5). Gadolinium was universally detectable in individuals with histories of magnetic resonance imaging contrast agent exposure. Inductively coupled plasma mass spectroscopy. *P* < 0.001 by Welch two sample *t-*test.

**Supplementary Fig. 14.** **Regression analysis for the United States Food & Drug Administration Event Reporting System (FAERS) reported gadolinium deposition disease (GDD) and nephrogenic systemic fibrosis (NSF) cases.** Using linear regression (linear models, adjusted r^2^), there was no correlation between ‘nephrogenic systemic fibrosis’ (**a**), or ‘gadolinium deposition disease’ (**b**) cases and log(*K_therm_*) or log(*K_cond_*). **c**, There is a linear correlation between NSF and GDD cases and years since approval (*P* = 0.002, adjusted *r^2^* = 0.7). ​

**Supplementary Fig. 15**. **Semithin renal sections for electron microscopy and XEDS.** **a,** Glutaraldehyde-fixed renal cortices embedded in resin were sectioned at 200 nm. Semithin sections were placed onto holey carbon coated 200 mesh Cu grids (SPI Supplies, West Chester, PA #3620C-MB, Lot# 1220811). **b,** Scanning transmission electron micrograph of proximal tubule using a high-angle annular dark field detector (1), provides sufficient contrast for the visualization of a proximal tubule nucleus (cyan outline), and organelles (magenta outlines) (2). Holey carbon mesh (yellow outline) (3) provides additional support for the delicate biological specimens. Calibration bar = 500 nm. JEOL 2010F FEGSTEM 200 kV transmission electron microscope, with Oxford Analytical AZTec XEDS system, equipped with XMax 80N 80 mm^2^ silicon drift detector.
