# Supplementary material for: The onset of rare earth metallosis begins with renal gadolinium-rich nanoparticles from magnetic resonance imaging contrast agent exposure
Source: Sci Rep. 2023 Feb 4;13:2025. doi: 10.1038/s41598-023-28666-1 (PMC9899216; doi:10.1038/s41598-023-28666-1)
Supplement: Supplementary file 17 — Supplementary Information 17. [file 41598_2023_28666_MOESM17_ESM.docx]

| **Supplementary Table 1. List of liver metabolites undergoing changes in expression resulting from magnetic resonance imaging contrast agent treatment.** {Capillary electrophoresis mass spectrometry (CE-MS), Human Metabolome Technologies (HMT, Japan). *P* values for liver metabolites from gadolinium-treated groups that differ from untreated liver were adjusted using the Benjamini-Hochberg (B-H) false discovery rate (FDR) correction.} | | | |
| --- | --- | --- | --- |
| **Metabolite** | **Pathway** | **Fold Change** | ***P* value** |
| 1-Aminocyclopropane-1-carboxylic acid | Amino Acid | 1.9 | 0.0002 |
| 1-Methyl-4-imidazoleacetic acid | Amino Acid | 1.4 | 0.00006 |
| 1-Methylhistamine | Amino Acid | 1.4 | 0.003 |
| 1-Pyrroline 5-carboxylic acid | Amino Acid | 1.2 | 0.02 |
| 2-Aminobutyric acid | Amino Acid | 1.1 | 0.03 |
| 2-Deoxyglucose 6-phosphate | Glycolysis | 1.1 | 0.0007 |
| 2-Hydroxy-4-methylvaleric acid | Amino Acid | 1.1 | 0.00006 |
| 2,6-Diaminopimelic acid | Amino Acid | 0.8 | 0.046 |
| Tryptophan | Amino Acid | -0.8 | 0.03 |
| Uridine Diphosphate (UDP) | Glycogenesis | -0.8 | 0.04 |
| Ala-Lys | Amino Acid | -1.2 | 0.04 |
| Glu-Cys | Amino Acid | -1.5 | 0.004 |
