# Supplementary material for: The onset of rare earth metallosis begins with renal gadolinium-rich nanoparticles from magnetic resonance imaging contrast agent exposure
Source: Sci Rep. 2023 Feb 4;13:2025. doi: 10.1038/s41598-023-28666-1 (PMC9899216; doi:10.1038/s41598-023-28666-1)
Supplement: Supplementary file 18 — Supplementary Information 18. [file 41598_2023_28666_MOESM18_ESM.docx]

| Supplementary Table 2. Energy-dispersive X-ray spectroscopy line scan data for subcellular regions of interest. {*P* values and adjusted *P* values for spectral signals from electron-dense precipitates, lipids, mitochondria, and other background regions using False Discovery Rate (FDR) with Benjamini-Hochberg procedure adjustment.} | | | | |
| --- | --- | --- | --- | --- |
| Region | **Comparator** | **Element** | ***P*** | ***Adjusted P*** |
| Precipitate | Other | Gd | 0 | 0 |
| Precipitate | Mitochondrion | Gd | 0 | 0 |
| Precipitate | Lipid | Gd | 0 | 0 |
| Precipitate | Other | Mg | 0 | 0 |
| Precipitate | Mitochondrion | Mg | 0 | 0 |
| Precipitate | Lipid | Mg | 0 | 0 |
| Precipitate | Other | P | 0 | 0 |
| Precipitate | Mitochondrion | P | 0 | 0 |
| Precipitate | Lipid | P | 3.95E-06 | 1.10E-05 |
| Precipitate | Other | Mn | 0 | 0 |
| Precipitate | Mitochondrion | Mn | 0 | 0 |
| Precipitate | Lipid | Mn | 0 | 0 |
| Precipitate | Other | Ca | 0 | 0 |
| Precipitate | Mitochondrion | Ca | 0 | 0 |
| Precipitate | Lipid | Ca | 7.70E-10 | 2.68E-09 |
| Precipitate | Other | S | 0 | 0 |
| Precipitate | Mitochondrion | S | 0.00052276 | 0.00120124 |
| Precipitate | Lipid | S | 0 | 0 |
| Precipitate | Other | N | 0.01939899 | 0.0317438 |
| Precipitate | Mitochondrion | N | 0.21599915 | 0.29528998 |
| Precipitate | Lipid | N | 0 | 0 |
| Precipitate | Other | Fe | 0 | 0 |
| Precipitate | Mitochondrion | Fe | 0.00029085 | 0.00069803 |
| Precipitate | Lipid | Fe | 0.69715369 | 0.80098509 |
| Precipitate | Other | Os | 2.81E-08 | 7.99E-08 |
| Precipitate | Mitochondrion | Os | 2.81E-08 | 7.99E-08 |
| Precipitate | Lipid | Os | 9.32E-06 | 2.40E-05 |
| Precipitate | Other | W | 0 | 0 |
| Precipitate | Mitochondrion | W | 0.00059929 | 0.0013484 |
| Precipitate | Lipid | W | 0.79206686 | 0.87711092 |
| Precipitate | Other | O | 0 | 0 |
| Precipitate | Mitochondrion | O | 9.68E-05 | 0.0002377 |
| Precipitate | Lipid | O | 0.015772 | 0.02661526 |
| Precipitate | Other | K | 8.65E-10 | 2.92E-09 |
| Precipitate | Mitochondrion | K | 0.03156436 | 0.05013164 |
| Precipitate | Lipid | K | 0.88573782 | 0.94712559 |
| Precipitate | Other | Zn | 0 | 0 |
| Precipitate | Mitochondrion | Zn | 0.0008508 | 0.00187524 |
| Precipitate | Lipid | Zn | 0.28661873 | 0.37749784 |
| Precipitate | Other | Cl | 1.16E-08 | 3.81E-08 |
| Precipitate | Mitochondrion | Cl | 0.81580038 | 0.88996405 |
| Precipitate | Lipid | Cl | 0.05184806 | 0.07886746 |
| Precipitate | Other | Mo | 0.16644493 | 0.23652701 |
| Precipitate | Mitochondrion | Mo | 0.44195474 | 0.54863347 |
| Precipitate | Lipid | Mo | 0.8785946 | 0.94712559 |
| Precipitate | Other | C | 0.20565937 | 0.28475913 |
| Precipitate | Mitochondrion | C | 0.57835487 | 0.69402585 |
| Precipitate | Lipid | C | 0.01272645 | 0.02216865 |
| Precipitate | Other | F | 0.00102963 | 0.00222399 |
| Precipitate | Mitochondrion | F | 0.63761116 | 0.74590509 |
| Precipitate | Lipid | F | 0.00406235 | 0.00783453 |
| Precipitate | Other | Si | 0.0055283 | 0.00995094 |
| Precipitate | Mitochondrion | Si | 0.99276935 | 0.99999888 |
| Precipitate | Lipid | Si | 0.00528675 | 0.00967744 |
| Lipid | Other | Gd | 4.93E-10 | 1.77E-09 |
| Lipid | Mitochondrion | Gd | 0.79589694 | 0.87711092 |
| Lipid | Other | Mg | 7.62E-06 | 2.01E-05 |
| Lipid | Mitochondrion | Mg | 0.59501226 | 0.7061684 |
| Lipid | Other | P | 0 | 0 |
| Lipid | Mitochondrion | P | 0.00125325 | 0.00265394 |
| Lipid | Other | Mn | 0 | 0 |
| Lipid | Mitochondrion | Mn | 0.04454659 | 0.06972509 |
| Lipid | Other | Ca | 0 | 0 |
| Lipid | Mitochondrion | Ca | 0.00280772 | 0.00561545 |
| Lipid | Other | S | 0.54014943 | 0.65546223 |
| Lipid | Mitochondrion | S | 0.77927427 | 0.87668355 |
| Lipid | Other | N | 7.85E-05 | 0.00019718 |
| Lipid | Mitochondrion | N | 0.04909561 | 0.07574751 |
| Lipid | Other | Fe | 0 | 0 |
| Lipid | Mitochondrion | Fe | 0.00342416 | 0.00672381 |
| Lipid | Other | Os | 2.81E-08 | 7.99E-08 |
| Lipid | Mitochondrion | Os | 2.81E-08 | 7.99E-08 |
| Lipid | Other | W | 0 | 0 |
| Lipid | Mitochondrion | W | 0.00473271 | 0.00896724 |
| Lipid | Other | O | 0.00030626 | 0.00071904 |
| Lipid | Mitochondrion | O | 0.05886874 | 0.08830311 |
| Lipid | Other | K | 0 | 0 |
| Lipid | Mitochondrion | K | 0.00656558 | 0.01162431 |
| Lipid | Other | Zn | 0 | 0 |
| Lipid | Mitochondrion | Zn | 4.22E-06 | 1.14E-05 |
| Lipid | Other | Cl | 0 | 0 |
| Lipid | Mitochondrion | Cl | 0.06225764 | 0.09210719 |
| Lipid | Other | Mo | 0.02078607 | 0.0335059 |
| Lipid | Mitochondrion | Mo | 0.19711502 | 0.27647302 |
| Lipid | Other | C | 0.71078456 | 0.80804981 |
| Lipid | Mitochondrion | C | 0.93005962 | 0.98476901 |
| Lipid | Other | F | 0 | 0 |
| Lipid | Mitochondrion | F | 0.00522658 | 0.00967744 |
| Lipid | Other | Si | 0.99996803 | 0.99999888 |
| Lipid | Mitochondrion | Si | 0.28044485 | 0.37392647 |
| Mitochondrion | Other | Gd | 0.01339936 | 0.02297032 |
| Mitochondrion | Other | Mg | 0.3113836 | 0.39564033 |
| Mitochondrion | Other | P | 0.00136815 | 0.00284154 |
| Mitochondrion | Other | Mn | 0.09147639 | 0.13172601 |
| Mitochondrion | Other | Ca | 0.30347316 | 0.39017978 |
| Mitochondrion | Other | S | 0.2938272 | 0.38232936 |
| Mitochondrion | Other | N | 0.99956183 | 0.99999888 |
| Mitochondrion | Other | Fe | 0.3980586 | 0.49988754 |
| Mitochondrion | Other | Os | 2.81E-08 | 7.99E-08 |
| Mitochondrion | Other | W | 0.09128266 | 0.13172601 |
| Mitochondrion | Other | O | 0.99999888 | 0.99999888 |
| Mitochondrion | Other | K | 0.50752533 | 0.62287199 |
| Mitochondrion | Other | Zn | 0.0016082 | 0.00327709 |
| Mitochondrion | Other | Cl | 0.01928991 | 0.0317438 |
| Mitochondrion | Other | Mo | 0.99714783 | 0.99999888 |
| Mitochondrion | Other | C | 0.99995678 | 0.99999888 |
| Mitochondrion | Other | F | 0.64230716 | 0.74590509 |
| Mitochondrion | Other | Si | 0.27307394 | 0.36864982 |
